# Griseolutein T from *Streptomyces seoulensis*, newly identified via combined-culture with *Tsukamurella pulmonis*, as an efficacious therapeutic agent against multidrug-resistant bacteria

**DOI:** 10.1038/s41429-025-00846-3

**Published:** 2025-07-09

**Authors:** Sung-Jin Kawai, Shumpei Asamizu, Hiroaki Suzuki, Hiroyasu Onaka, Yoshichika Arakawa, Kouji Kimura, Makoto Ojika

**Affiliations:** 1 New Field Pioneering Division, Toyota Boshoku Corporation, Kariya, Aichi Japan; 2 Graduate School of Agricultural and Life Sciences, The University of Tokyo, Tokyo, Japan; 3 Collaborative Research Institute for Innovative Microbiology, The University of Tokyo, Tokyo, Japan; 4 Engineering Biology Research Center, Kobe University, Kobe, Japan; 5 Department of Life Science, Faculty of Science, Gakushuin University, Tokyo, Japan; 6 Graduate School of Medicine, Nagoya University, Nagoya, Japan; 7 Graduate School of Bioagricultural Sciences, Nagoya University, Nagoya, Japan; 8 Present Address: Department of Microbiology, Fujita Health University School of Medicine, Toyoake, Aichi Japan

**Keywords:** Antibiotics, High-throughput screening

## Abstract

Bacterial interactions can affect the production of secondary metabolites and, therefore, provide a promising approach to exploring new microbial compounds. In this study, we screened actinomycetes isolated from Hegura Island, Ishikawa Prefecture, Japan, to discover new antibiotics through combined-culture with *Tsukamurella pulmonis* TP-B0596. Three new phenazine-class antibiotics, griseoluteins T (**1**), C (**2**), and D (**3**), along with two known related metabolites, griseoluteic acid (**4**) and griseolutein A (**5**), were detected in both mono- and combined-cultures of *Streptomyces seoulensis* HEK131 with *T. pulmonis* at different production levels. Detailed spectroscopic analysis revealed that **1** contained a dihydrophenazine core, and was converted to **5** by accepting oxidation spontaneously. **1**, containing a dihydrophenazine group, was relatively unstable under oxidative conditions, and the addition of ascorbate was required during the isolation of the compound. **2** and **3** were found to be cysteine-adducts analogous to **4**, and their productivity was increased in the combined-culture. We further assessed the antibacterial activities of **1** against clinically significant Gram-positive pathogenic bacteria, including 30 methicillin-resistant *Staphylococcus aureus* (MRSA), 27 vancomycin-resistant *Enterococci* (VRE), and 17 *Clostridioides difficile*. Notably, **1** was found to possess higher antibacterial activity against these microorganisms than several clinically important antibiotics, while displaying lower cytotoxicity against HeLa-S3 cells.

## Introduction

Filamentous actinomycetes are the source of more than 60% of the known natural product antibiotics [1]. The genomes of actinomycetes have been found to contain a number of gene clusters for secondary metabolite biosynthesis, including pharmaceutically important compounds [2, 3]. However, most of the biosynthetic gene clusters encoding secondary metabolites are silent in culture under normal laboratory conditions [4]. Bacterial co-culture has been found to activate the laboratory silent biosynthetic gene clusters for the production of secondary metabolites in Actinobacteria [5–9].

Combined-cultures, comprising a specific combination of actinomycetes and mycolic acid-containing bacteria, including *Tsukamurella pulmonis* TP-B0596, have been successful in augmenting the production of cryptic secondary metabolites in monocultures, resulting in the isolation of 44 new compounds from 15 actinomycetes to date [5, 10, 11]. In combined-cultures, modifications of known compounds have also been observed. These include harundomycin A, a conjugate between platensimycin and enterobactin from *Streptomyces hygroscopicus* HOK021 [11]; desferrioxamines modified with an unusual N-containing five-membered ring structure, termed I2a, I1a, and I1b, from *Streptomyces davawensis* JCM 4913 [12]; conversion of [5,5]-spirohemiaminals to 5-alkyl-1,2,3,4-tetrahydroquinolines in *Streptomyces nigrescens* HEK616, which may involve an additional oxidation step [13]; and variations in the cyclization patterns of macrolactams from similar backbones, such as sceliphrolactam to niizalactam [14], micromonolactam to dracolactam [15], and mirilactam A to mirilactam C, D, and E [16], which may involve additional oxidative pathways. Mechanisms of activation in combined-culture for secondary metabolism have been investigated using model actinomycetes [17–20], which may involve as yet unidentified mechanisms along with multiple cellular processes.

Multidrug-resistant bacteria present an urgent and serious public health threat. It has been estimated that the number of deaths due to infectious diseases caused by antimicrobial resistance will exceed 10 million per year by 2050 [21]. In fact, infectious disease mortality due to multidrug-resistant bacteria is increasing year by year [22]. The development of novel antibiotics that operate via new antibacterial mechanisms is a promising approach to combat this increasing threat.

We previously isolated 854 actinomycetes from environmental samples collected at Hegura Island, Ishikawa Prefecture, Japan [23], and discovered that combined-culture successfully induced the specific production of various metabolites [15, 24, 25]. To search for new antibiotics active against clinically hazardous drug-resistant bacteria, we performed mono- and combined-culture of the actinomycetes strains with *T. pulmonis* and tested their antibacterial activities. A bioassay-guided fractionation of the culture broths of a selected *Streptomyces seoulensis* HEK131 strain resulted in the discovery of promising metabolites with a phenazine core structure. Here, we report the structural elucidation and biological activities of the isolated metabolites.

## Materials and methods

### General

Actinomycetes and *T. pulmonis* were cultured using ISP2 and A3M media. ISP2 agar medium contained glucose (0.4%), malt extract (1.0%, Difco, BD Biosciences, CA, USA), yeast extract (0.4%, Bacto, BD Biosciences), and agar (1.5%) in distilled water (pH 7.2). A3M medium contained starch (2%), glucose (0.5%), glycerol (2%), pharmamedia (1.5%), yeast extract (0.3%), and Diaion HP-20 (1%, pH 7.0). Cultures were grown in 96-well plates, shaken on a Maximizer MBR-420FL (TAITEC, Tokyo, Japan). Plates were sealed with breathable film (SureSeal RA Breathable; BMBio, Tokyo, Japan) during cultivation. For butanol extraction, plates were sealed with an aluminum seal (WATSON, Tokyo, Japan). Solvents were removed using a centrifugal concentrator equipped with a cold trap (EC500 and CS-110-4, respectively; Sakuma Co., Tokyo, Japan).

### Instruments and settings

Optical rotations were recorded on a DIP-370 digital polarimeter (Jasco, Tokyo, Japan). UV and IR spectra were measured on a V-730 BIO spectrophotometer and an FT/IR-4100 (Jasco), respectively. NMR spectra were recorded on an Avance ARX400 (400 MHz for ^1^H, 100 MHz for ^13^C) or an Avance III HD 600 MHz Cryo-probe spectrometer (600 MHz for ^1^H, 150 MHz for ^13^C) (Bruker Japan, Yokohama, Japan) using the residual solvent peak of DMSO-*d*_6_ at *δ* 2.50 for ^1^H and *δ* 39.5 for ^13^C as internal standards. Preparative high-performance liquid chromatography (HPLC) was performed on a high-pressure gradient system equipped with PU-2087plus pumps, a DG-2080-53 degasser, and a UV-2075plus detector (Jasco). LC/MS analysis was performed by a 6520 Accurate-Mass Q-TOF spectrometer (Agilent Technologies, Santa Clara, CA, USA) in the electrospray ionization mode (positive) and a 1100 HPLC system (Agilent Technologies).

### Bacterial strains used in this study

Eight hundred and fifty-four strains of actinomycetes, collected from Hegura Island, Ishikawa Prefecture in Japan, were used for mono- and combined-culture with *Tsukamurella pulmonis* TP-B0596. Methicillin-susceptible *Staphylococcus aureus* ATCC29213 (MSSA), methicillin-resistant *S. aureus* ATCC43300 (MRSA), *Escherichia coli* ATCC25922, *E. coli* ATCC BAA-2471 (NDM-1-producing reference strain), *Enterococcus faecalis* ATCC51299 (*vanB* positive, VRE), and *Clostridioides difficile* ATCC700057 were purchased from American Type Culture Collection for the antibacterial activity tests. *E. coli* E1346 is a clinically isolated strain with resistance to colistin as it harbors the *mcr-*1 gene [26]. Colistin and ampicillin were purchased from FUJIFILM Wako Pure Chemical (Tokyo, Japan) and used as controls.

### Culture plate screening of antibacterial compounds

For screening using 96-deep-well culture plates, first, *T. pulmonis* was streaked onto ISP2 agar medium and cultivated for 2 days at 30 °C. Then, for the seed culture, *T. pulmonis* was cultivated in a test tube containing 10 mL of ISP2 medium, which was shaken on a rotary shaker (200 rpm) at 30 °C for 2 days. Each frozen stock of actinomycetes (854 strains) was inoculated separately into a well of a 96-deep-well plate containing 100 µL of ISP2 medium for seed culture. The plates were shaken on a Maximizer (1500 rpm) at 30 °C for 3 days for the pre-culture. Then, for the production combined-culture, A3M medium (500 µL) with 1.5% (v/v) of *T. pulmonis* culture broth was added to the 96-deep-well plate containing the actinomycetes seed cultures. For monoculture, only A3M medium (500 µL) was added. Then, all plates were sealed with breathable seal and shaken on the Maximizer (1500 rpm) at 30 °C for 3 days or 7 days. To minimize evaporation of the culture medium, the outer 36 wells of a 96-well plate were filled with water, and cultivation was conducted in the inner 60 wells (arranged in 6 rows × 10 columns). Although evaporation could not be completely prevented, the remaining culture medium was used directly for butanol extraction. After cultivation, 500 µL of butanol was added to each well, sealed with an aluminum seal, and shaken on the Maximizer (1500 rpm) at 30 °C for 30 min for extraction. After centrifugation (3000 rpm, 10 min, room temperature), the supernatants (butanol layers) were transferred into a new microplate and dried *in vacuo* using a centrifugal concentrator equipped with a cold trap. The dried crude extracts were dissolved in 0.1 vol. of dimethyl sulfoxide (DMSO) and transferred to new microplates for an antibiotic assay.

### Extraction and isolation of active compounds from *S. seoulensis* HEK131

A 100 mL broth of *S. seoulensis* HEK131 (monoculture or combined-cultured with *T. pulmonis*) was diluted with EtOH (100 mL), and sodium ascorbate (10 mg) was added to prevent the autooxidation of compounds, because griseolutein T (1) was found to be susceptible to autoxidation. The oxidation product was formed without the addition of sodium ascorbate prior to the separation process.

The mixture was shaken at 180 rpm for 1 h and centrifuged at 6000 rpm for 10 min to extract the metabolites. The supernatant was separated, and the precipitate was extracted again with 50% EtOH (20 mL). The first supernatant and the second cell extracts were combined and concentrated to approximately 30 mL. A portion (0.1%) of the extract was stored and used for LC/MS analysis. Two extracts obtained from the 5-day cultures without *T. pulmonis* (200 mL in total) were combined and chromatographed on an octadecylsilyl (ODS) silica gel column (Cosmosil 75C_18_-PREP, 500 g, Nacalai Tesque, Kyoto, Japan) eluted with 20%, 30%, 40%, and 50% acetonitrile (MeCN) in H_2_O containing 0.1% trifluoroacetic acid and 0.01% sodium ascorbate (250 mL × 4 for each fraction batch). Fractions 7 and 8 eluted with 30% MeCN were combined and concentrated to give a brown material-I (229 mg). Material-I was purified by preparative HPLC [Develosil ODS-HG-5 (20 × 250 mm, Nomura Chemical, Aichi, Japan), 10%–40% MeCN-20 mM NH_4_OAc, 60 min linear gradient, 6 mL min^−1^, detected at 273 nm] to give **1** (1.04 mg, *t*_R_ = 24.7 min). Fractions 11–15 eluted with 40%–50% MeCN were combined and concentrated to give a brown material-II (250 mg). Material-II was purified by preparative HPLC [Develosil ODS-HG-5 (20 × 250 mm), 25%–35% MeOH-20 mM NH_4_OAc, 60 min linear gradient, 5 mL min^−1^, detected at 265 nm] to give **2** (0.05 mg, *t*_R_ = 57.3 min) and **4** (0.03 mg, *t*_R_ = 50.7 min).

To further obtain the purified compounds, the extracts obtained from the 4-, 5-, and 6-day combined-culture broths (300 mL in total) were combined, extracted, and chromatographed on an ODS column under the same conditions described above. Fractions 7 and 8 eluted with 30% MeCN were combined and concentrated to give a brown material-III (245 mg). Material-III was purified by HPLC under the same conditions as material-I to obtain another purified **1** (0.83 mg, *t*_R_ = 25.0 min). Fractions 10–14 eluted with 40%–50% MeCN were combined and concentrated to give a brown material-IV (290 mg). Material-IV was purified by HPLC (under the same conditions as material-II) to obtain another purified **2** (0.52 mg, *t*_R_ = 58.1 min), **3** (0.17 mg, *t*_R_ = 39.2 min), and **4** (0.03 mg, *t*_R_ = 48.5 min). Some samples containing oxidized **1** were purified by HPLC (under the same conditions as material-I) to give **5** (0.08 mg, *t*_R_ = 35.1 min).

Griseolutein T (1): UV λ^MeOH^_max_ nm (ε) 224 (23,000, sh), 267 (14,300), 341 (5700); IR (KBr) cm^−1^ 3385, 1677, 1607, 1588, 1510, 1478, 1446, 1397, 1287, 1260, 1105, 1026, 999, 768; NMR (see Table 1), ESI-TOF MS *m/z* 327.0973 (calcd for C_17_H_15_N_2_O_5_ [M − OH]^+^ 327.0975), 343.0916 (calcd for C_17_H_15_N_2_O_6_ [M − H]^+^ 343.0925), 367.0892 (calcd for C_17_H_16_N_2_O_6_Na [M + Na]^+^ 367.0901).

**Table 1:** NMR data for griseolutein T (1), C (2), and D (3) (in DMSO-*d*_6_)^a^

No	Griseolutein T (1)		Griseolutein C (2)		Griseolutein D (3)	
	*δ*_C_	*δ*_H_ (mult., *J* in Hz)	*δ*_C_	*δ*_H_ (mult., *J* in Hz)	*δ*_C_^*d*^	*δ*_H_ (mult., *J* in Hz)
1	125.2	-	127.2^*d*^	-	129.1	-
2	129.4	7.77 (d, 7.6)	134.8	8.63 (d, 6.8)	133.4	8.49 (d, 8.8)
3	118.2	6.84 (t, 7.6)	131.0	8.12 (dd, 8.4, 6.8)	130.4	8.08 (dd, 8.0, 7.2)
4	126.1	7.36 (d, 7.6)	133.8	8.53 (d, 8.4)	133.6	8.48 (m)
4a	123.0	-	141.8^*e*^	-	141.7	-
5a	123.3	-	141.9^*e*^	-	141.5	-
6	130.7	-	128.8	-	138.0	-
7	118.6	7.04 (d, 8.4)	131.3	7.94 (d, 8.0)	130.8	8.01 (m)
8	108.6	6.90 (d, 8.4)	108.9	7.40 (d, 8.0)	132.4	8.02 (m)
9	145.1	-	152.5	-	127.7	8.27 (m)
9a	129.4	-	133.2	-	141.1	-
10a	142.7	-	137.9	-	139.6	-
1’	59.1	4.42, 4.45 (d, 14.6)	29.9	4.35, 4.40 (d, 13.6)	30.0	4.47, 4.53 (d, 13.2)
1”	n.d.^*b*^	-	172.0	-	172.1	-
2”	59.8	3.64, 4.37 (brd, 13.2)	52.6	4.37 (m)	52.2	4.44 (m)
3”	-		33.7	2.76 (dd, 13.5, 7.4) 2.94 (dd, 13.5, 4.8)	33.4	2.78 (dd, 13.6, 8.0) 2.95 (dd, 13.6, 5.0)
1-CO	169.5	-	165.9	-	166.5	-
9-OMe	56.0	3.84 (s)	56.5	4.12 (s)	-	-
1’-OH	-	4.91 (brs)^*c*^	-	-		-
2”-OH	-	4.73 (brt)^*c*^	-	-		-
NH	-	11.54 (s)	-	7.98 (m)		8.10 (m)
Ac	-	-	168.9, 22.5	1.83 (s)	169.1, 22.4	1.84 (s)

^a^Measured at 400 MHz for ^1^H and 100 MHz for ^13^C

^b^Not detected

^c^Detected in another spectrum measured at 600 MHz

^d^Determined by HMBC or heteronuclear single quantum coherence (HSQC)

^e^Interchangeable signals

Griseolutein C (2): [α]^14^_D_ –6 (*c* 0.016, MeOH), UV λ^MeOH^_max_ nm (ε) 247 (31,600), 273 (41,700), 370 (13,500); IR (KBr) cm^−1^ 3413, 3079, 1716, 1653, 1602, 1542, 1465, 1286,1134, 1096, 1025, 766; NMR (see Table 1), ESI-TOF MS *m/z* 430.1068 (calcd for C_20_H_20_N_3_O_6_S [M + H]^+^ 430.1067), 452.0878 (calcd for C_20_H_19_N_3_O_6_SNa [M + Na]^+^ 452.0887).

Griseolutein D (3): [α]^14^_D_ –32 (*c* 0.031, MeOH), UV λ^MeOH^_max_ nm (ε) 253 (60900), 370 (15100); IR (KBr) cm^−1^ 3425, 1715, 1649, 1538, 1465, 1026, 999, 761; NMR (see Table 1), ESI-TOF MS *m/z* 400.0958 (calcd for C_19_H_18_N_3_O_6_S [M + H]^+^ 400.0962), 422.0777 (calcd for C_19_H_17_N_3_O_6_SNa [M + Na]^+^ 422.0781).

Griseoluteic acid (4) [27, 28]: ^1^H NMR (400 MHz, DMSO-*d*_6_) *δ* 8.55 (1H, brd, *J* = 8.0 Hz), 8.48 (1H, d, *J* = 7.6 Hz), 8.12 (1H, m), 7.97 (1H, d, *J* = 8.0 Hz), 7.45 (d, *J* = 8.0 Hz), 5.20 (2H, s), 4.12 (3H, s); ESI-TOF MS *m/z* 285.0867 (calcd for C_15_H_13_N_2_O_4_ [M + H] ^+^ 285.0870) (See Supplementary Table S1 for the comparison with reported values).

Griseolutein A (5) [29]: ^1^H NMR (400 MHz, DMSO-*d*_6_) *δ* 8.22 (1H, d, *J* = 8.0 Hz), 8.05 (1H, m), 7.97 (1H, m), 7.94 (1H, d, *J* = 8.0 Hz), 7.33 (1H, d, *J* = 8.0 Hz), 5.80 (2H, s), 5.39 (1H, m), 4.11 (3H, s), 4.09 (2H, m); ESI-TOF MS *m/z* 343.0905 (calcd for C_17_H_15_N_2_O_6_ [M + H] ^+^ 343.0925).

### Antibacterial activity test

*E. coli* BAA-2471 (NDM-1-producing strain), *E. coli* E1346 (MCR-1-producing strain), *E. coli* ATCC25922, and *S. aureus* (MRSA) were used for an antibiotic assay. MRSA was cultivated overnight at 30 °C, and other *E. coli* strains were cultivated overnight at 37 °C. Colonies of each strain were suspended in saline and adjusted to McFarland 0.5 by a MicroScan Turbidity Meter (Siemens, Bayern, Germany). Then, the adjusted suspension was diluted (200×) with Mueller Hinton II Broth (MHB, cation-adjusted) (Nippon Becton Dickinson Company, Ltd, Tokyo, Japan). In each well of the microtiter plate, a crude extract in DMSO solution (10 μL) was added to 200 μL of bacterial suspension diluted with MHB. The plates were incubated at 37 °C or 30 °C for 16–20 h. Ampicillin and colistin were used as positive controls.

ATCC strains of *S. aureus* (MRSA), *S. aureus* (MSSA), *E. faecalis*, and *C. difficile* were used as standard strains. Clinical isolates of MSSA (30 isolates), MRSA (30 isolates), VRE (27 isolates), and *C. difficile* (17 isolates) were supplied by the Department of Bacteriology, Nagoya University School of Medicine (Supplementary Tables S2–S6) and used in an antimicrobial susceptibility test. MIC_50_ values were determined by the microdilution method according to the CLSI guidelines [30]. The following commercial antibiotics were selected for comparison of the MIC_50_ values with compound **1**. Vancomycin (FUJIFILM Wako Pure Chemical Corporation, Tokyo, Japan), which is one of the anti-MRSA drugs; fidaxomicin (Tokyo Chemical Industry Co., Ltd [TCI], Tokyo, Japan), which is one of the drugs used to treat *C. difficile* infections; tigecycline (TCI, Tokyo, Japan), which is used to treat Gram-negative and -positive bacterial infections; mupirocin (TCI, Tokyo, Japan), which is used as a topical treatment for MRSA infections; and phenazine-1-carboxylic acid (tubermycin B; Toronto Research Chemicals, Toronto, Canada) as an analog of compound **1**.

Ascorbate was used as an additive during the purification stage to ensure an effective yield of **1**. However, because such additives are not appropriate for biological assays, the bioactivity of **1** was evaluated with consideration of its inherent instability.

### Cytotoxicity assay

The HeLa-S3 cells were obtained from RIKEN BRC (Tsukuba, Ibaraki, Japan). The medium consisted of Eagle’s Minimal Essential Medium (445 mL) (EMEM, FUJIFILM Wako Pure Chemical, Osaka, Japan), fetal bovine serum (50 mL) (heat inactivated, Thermo Fisher Scientific, Waltham, MA, USA), and an antibiotic mix (5 mL) (10,000 units mL^−1^ penicillin, 10,000 µg mL^−1^ streptomycin, Thermo Fisher Scientific). The cells (1 × 10^4^ cells per well) in 99 µL of the medium were incubated in the wells of a 96-well plate at 37 °C for 24 h in a 5% CO_2_ incubator (MCO-96, Sanyo Electric, Osaka, Japan). Then, 1 µL of a compound in 50% DMSO, staurosporine (Fujifilm, Osaka, Japan) or paclitaxel (Fujifilm) in 50% DMSO as positive controls, or 50% DMSO as the negative control, was added to each well. After incubating for 48 h, the cells were treated with 10 µL of 3-(4,5-dimethylthiazol-2-yl)-2,5-diphenyltetrazolium bromide (MTT) in phosphate buffer saline (5 mg mL^−1^) and incubated for an additional 3 h. The medium was removed, and the cells were dissolved in 100 µL of DMSO and shaken for 5 min. Finally, the absorbance values were measured at 595 nm using a Multiscan FC microplate reader (Thermo Fisher Scientific). The IC_50_ values were calculated by sigmoid fitting curves generated from the mean values from three experiments at each concentration. Because additives such as ascorbate are not suitable for biological assays, the bioactivity of **1** was evaluated with consideration of its inherent instability.

## Results and discussion

### Screening of actinomycetes producing antibacterial compounds

The 854 previously reported actinomycete strains were isolated from Hegura Island, Ishikawa Prefecture, Japan [23]. They were grown in both mono- and combined-cultures with *T. pulmonis* using 96-well culture plates, and the butanol extracts were used for the antibacterial assay. As a result, extracts of combined-culture from 37 strains (out of the 854 strains) showed more than 10% growth inhibition specifically against three drug-resistant bacteria: a Gram-negative *Escherichia coli* strain BAA-2471 (NDM-1) exhibiting carbapenem resistance, an *E. coli* strain E1346 (MCR-1) exhibiting colistin resistance, and a Gram-positive methicillin-resistant *Staphylococcus aureus* (MRSA) strain ATCC43300 exhibiting multidrug resistance. We selected these 37 actinomycete strains for large-scale culture. When the culture volume was scaled up to flask culture (100 mL in a K-1 flask), seven strains (out of the 37 strains) showed consistent antimicrobial activity against the three drug-resistant bacteria tested. We first isolated the active metabolites against *E. coli* strains from these seven strains. However, most of their growth inhibition activities against *E. coli* strains were found to be derived from nocardamine, a siderophore well-known as an iron-chelating natural product (data not shown) [23, 31]. We then sought active metabolites against MRSA. Among the seven strains, the crude extracts from *Streptomyces seoulensis* HEK131 exhibited strong antibacterial activity against MRSA. After chromatographic separation (Supplementary Figs. S1 and S2), the metabolites in the active fraction were found to contain unknown chemical properties. We therefore isolated these metabolites for detailed structural elucidation.

### Discovery of bioactive phenazine metabolites from *S. seoulensis* HEK131

**Fig. 1:**
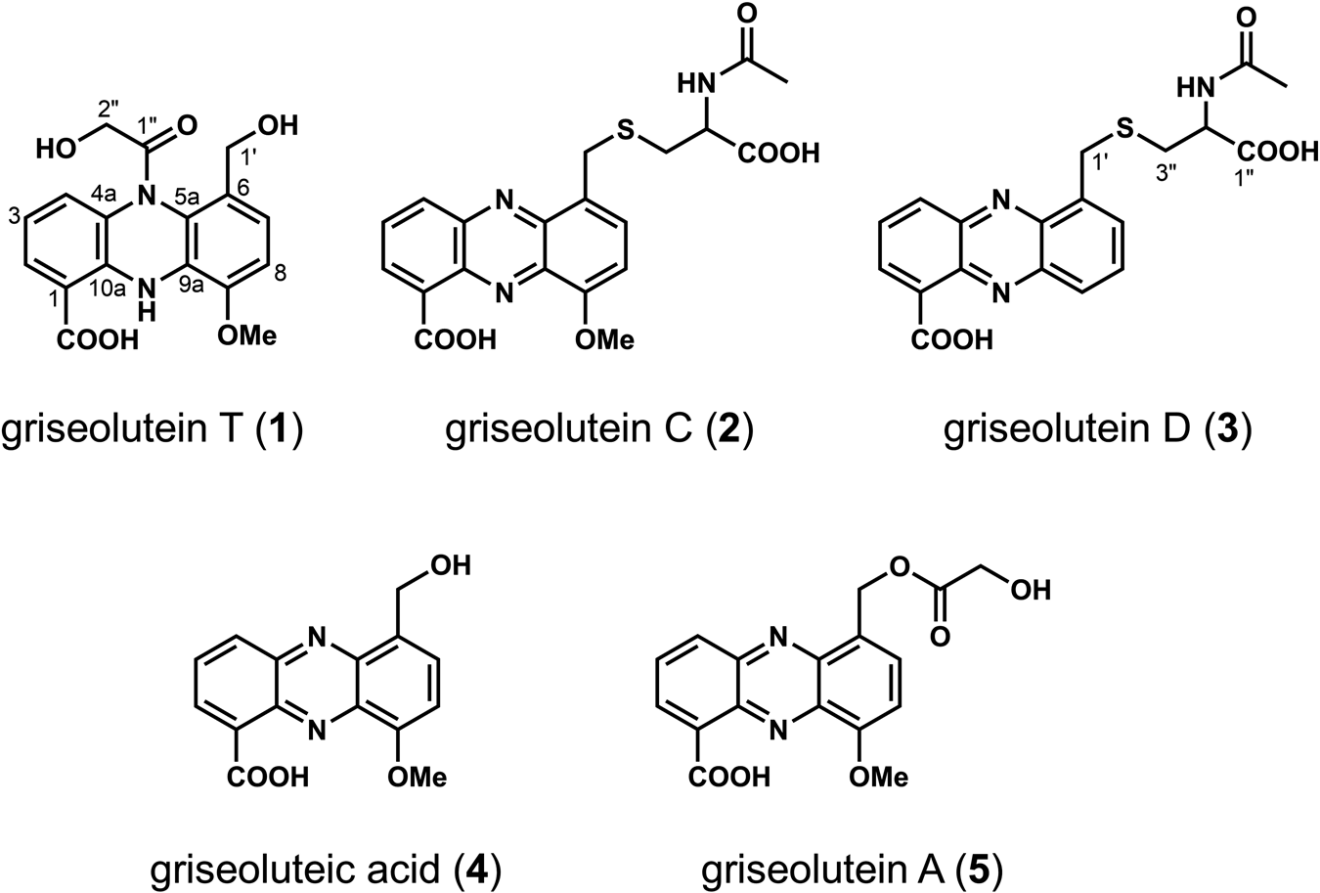
Structures of griseolutein T (**1**), griseolutein C (**2**), griseolutein D (**3**), and known related metabolites griseoluteic acid (**4**) and griseolutein A (**5**) isolated in this study

*S. seoulensis* HEK131 was grown in a 100 mL culture for antibiotic production, and the active compounds were isolated and identified as described in the Materials and Methods section (Supplementary Fig. S1 and S2). Five compounds [griseoluteins T (1), C (2), D (3), griseoluteic acid (4), and griseolutein A (5), Fig. 1] were isolated to homogeneity and the chemical structures were predicted using high-resolution mass spectrometry and a series of nuclear magnetic resonance (NMR) spectra (described later). Notably, among the isolated metabolites, **1** was found to be susceptible to oxidative transformation after several rounds of purification. Therefore, the chromatographic processes were performed in the presence of sodium ascorbate, except for the final purification, to prevent autoxidation.

### Structural analysis of griseolutein T (1) isolated from *S. seoulensis* HEK131

Griseolutein T (1) has the molecular formula C_17_H_16_N_2_O_6_ based on the exact ion masses determined by an electrospray ionization time-of-flight mass spectrometer (ESI-TOF-MS). The ions of *m/z* 327.0973 (obs.) and 367.0892 (obs.) were assigned as [M − OH]^+^ (calcd 327.0975) and the sodium adduct form [M + Na]^+^ (calcd 367.0901), respectively, of **1** (Supplementary Fig. S3). Another ion of *m/z* 343.0916 (obs.) corresponding to [M − H]^+^ (calcd 343.0925) was observed (Supplementary Fig. S3). Observation of this non-typical ion may indicate that **1** is susceptible to oxidation (dehydrogenation) to form the known metabolite griseolutein A (5) (C_17_H_14_N_2_O_5_). Taken together, **1** was predicted to be a dihydro analog of **5**.

**Fig. 2:**
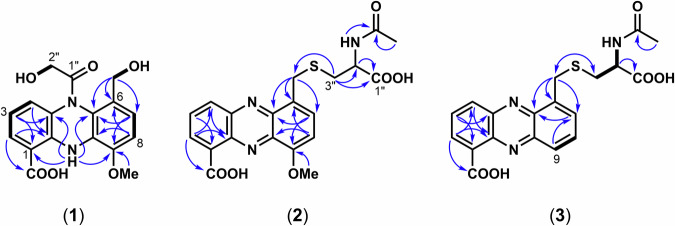
Selected COSY (thick bonds) and HMBC correlations (arrows) for **1**, **2**, and **3**

The structure of **1** was then analyzed by NMR experiments (Supplementary Figs. S4–S8). A correlation spectroscopy (COSY) experiment revealed two unsaturated hydrocarbon structures, =CH–CH = CH– and –CH = CH– (Fig. 2). Other detected substructures were two hydroxymethyl groups and one methoxy group. A notable ^1^H signal was observed at *δ* 11.54 (s), which was estimated to be NH based on the predicted molecular formula. The ^13^C NMR spectrum indicated the presence of two carbonyls and seven *sp*^2^ quaternary carbons. These partial structures were finally connected by a heteronuclear multiple bond correlation (HMBC) experiment (Fig. 2). Although the HMBC correlation between H-2” and C-1” of the hydroxyacetyl group was not observed, the presence of the group and its position were automatically determined from the molecular formula.

**Fig. 3:**
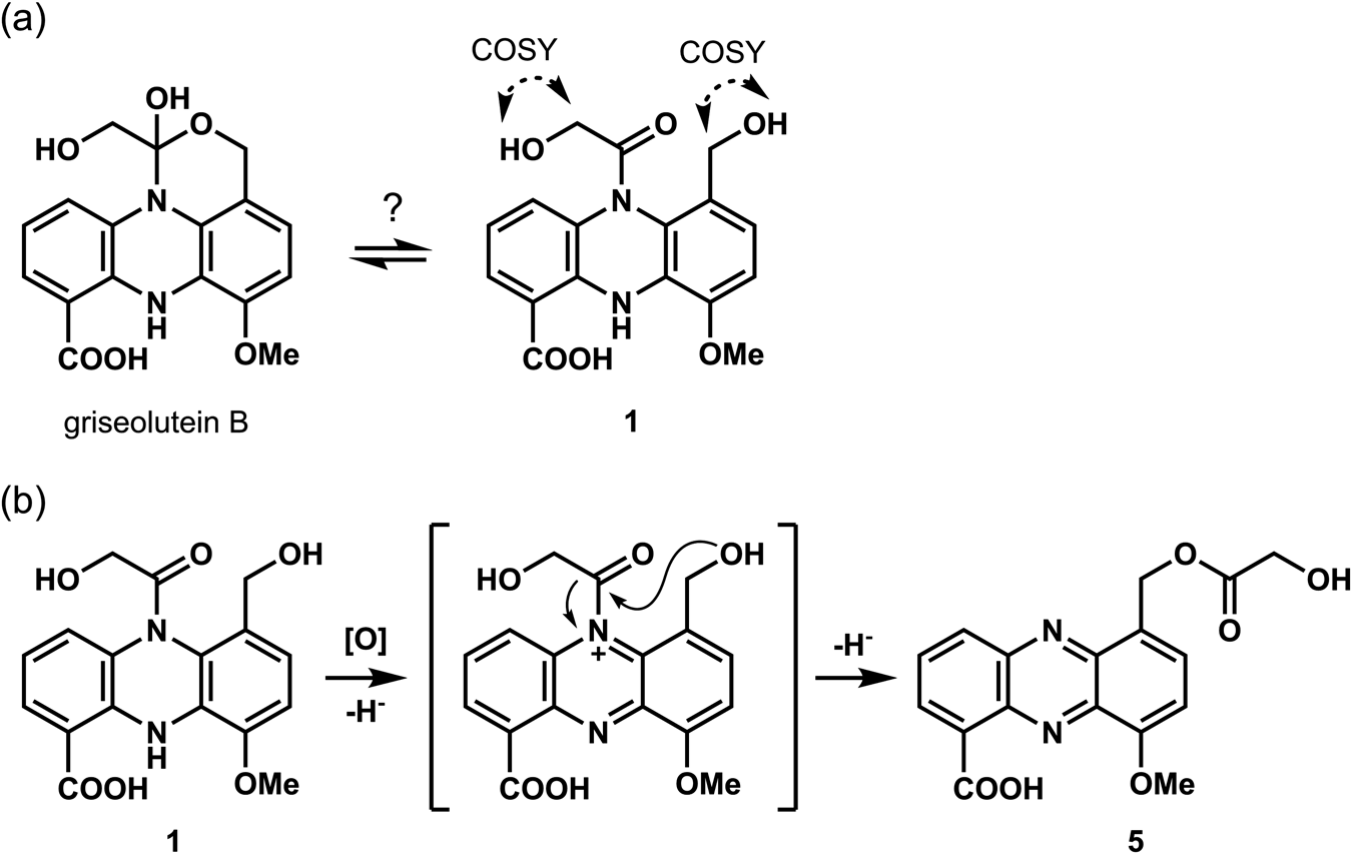
Proposed structure of griseolutein B and its structural relationship to **1** (**a**), and plausible mechanism of the conversion of **1**–**5** (**b**)

The structure of griseolutein B [32], which was revised in 1964 [33], was analogous to that of **1** (Fig. 3a). The NMR of **1** in our study showed a similar spectrum to that of griseolutein B, which is a cyclized isomer of **1** (Fig. 3a). Since we observed clear COSY correlations between the protons of two hydroxy groups and each connected methylene (2″ and 1′) (Fig. 2), it was possible to discriminate **1** from griseolutein B (Fig. 3a). Although a theoretically proposed identical structure has been reported in the literature [34], we believe that this is the first study to perform isolation and provide spectral evidence to reveal the chemical structure of **1**.

**1** was susceptible to autoxidation to form **5**, which was the first griseolutein antibiotic to be discovered in 1959 [29]. This instability is due to the dihydrophenazine structure. We predicted that **1** first undergoes hydride removal to form an acyl ammonium ion intermediate followed by an intramolecular acyl shift to the 1′-OH group, resulting in the aromatization of the tricyclic system (Fig. 3b). In conclusion, we believe that **5** may be an oxidized shunt product of **1**, and that **1** is a true biosynthetic metabolite. Based on its chemical properties, **1** may play some role in the redox system, as predicted for the phenazine-class antibiotic, pyocyanine from *Pseudomonas aeruginosa* [35].

### Structural analysis of griseolutein C (2) and D (3) isolated from the combined-culture of *S. seoulensis* HEK131 and *T. pulmonis*

The molecular formula of griseolutein C (2) was determined as C_20_H_19_N_3_O_6_S by HR ESI-TOF MS (Supplementary Fig. S3). The M + 2 isotopic peak intensity of **2** was theoretically consistent with the natural isotope pattern of sulfur, suggesting the presence of a sulfur atom in the molecule (Supplementary Table S7). The NMR spectrum (Table 1, Supplementary Figs. S9–S12) showed similarity to that of **1**, but significant differences were the lack of the hydroxyacetyl group and the low-field NH signal in **1**, and the presence of an extra substructure that contains a –CH_2_–CH< system. The ^1^H–^13^C long-range connectivity was determined by an HMBC experiment (Fig. 2, Supplementary Fig. S12), revealing a novel griseolutein-type structure for **2**, which contains a cysteine substructure. The molecular formula of griseolutein D (3) was determined as C_19_H_17_N_3_O_5_S by HR ESI-TOF MS (Supplementary Fig. S3). The M + 2 isotopic peak intensity of **3** was theoretically consistent with the natural isotope pattern of sulfur, suggesting the presence of a sulfur atom in the molecule (Supplementary Table S7). The NMR data (Table 1, Supplementary Figs. S13–S17) were similar to those of **2**, except for the lack of the methoxy group at the C-9 position. Although ^13^C data were inadequate due to low yield, the structure was determined by its limited 2D NMR correlations (Fig. 2) and comparison of the chemical shifts between **2** and **3**. Griseoluteic acid (4) was isolated as a minor component, and the structure was determined by comparison with the reported MS and ^1^H NMR data (Supplementary Table S1) [32].

Using LC/MS, the amounts of metabolites **1**–**5** were quantified to analyze the production profiles in mono- and combined-culture (Supplementary Fig. S18). The standard curves of each metabolite [1–5] for quantification were generated using the isolated metabolites. In the monoculture from day 5, **2** was undetectable, and the yield of **3** was 2.3 mg L^−1^. By contrast, in the combined-culture from day 5, the yield of **2** was 1.5 mg L^−1^ and the yield of **3** was 11 mg L^-1^, indicating the increased productivity of **2** and **3** in the combined-culture. Similar to compounds **2** and **3**, cysteinylated streptophenazines have also been isolated from *Streptomyces* sp. ID63040 [36]. It is speculated that the formation of cysteinylated streptophenazines occurs through the addition of mycothiol, which is used to maintain cellular redox balance and is known to play a role in detoxifying xenobiotics [37]. In combined-culture, it is hypothesized that these cellular processes were enhanced, leading to the increased production of compounds **2** and **3**. Although **4** was originally reported as a degradation product of griseolutein B [38], it was subsequently detected in the culture broth of *S. griseoluteus* and regarded as a biosynthetic precursor of griseoluteins based on the observation that **4** was converted to **5** and griseolutein B following treatment with cell homogenates [28].

### Antibacterial activities and cytotoxicity of isolated compounds

Cytotoxicity (IC_50_) against the HeLa-S3 cell line was evaluated for **1**–**5** using the MTT assay (Table 2, Supplementary Fig. S19). **1** (1.6 µM) exhibited the highest potency, followed by **5** (2.5 µM) and **4** (8.1 µM). By contrast, the cysteine-bound analogs **2** and **3** showed lower cytotoxicity (96 and 78 µM, respectively). Staurosporine and paclitaxel were included as positive controls. Compared with these standards, **1** showed lower cytotoxicity (30-fold lower than staurosporine and 170-fold lower than paclitaxel).

**Table 2:** Cytotoxicity of 1–5 against HeLa-S3 cells

Compounds	1	2	3	4	5	STA	PAC
IC_50_ (µM)	1.6	96	78	8.1	2.5	0.049	0.0094

*STA* staurosporine, *PAC* paclitaxel. STA and PAC were generally used as the positive controls

Four clinically isolated Gram-positive bacterial strains—methicillin-susceptible *S. aureus* (MSSA), methicillin-resistant *S. aureus* (MRSA), vancomycin-resistant *Enterococci* (VRE), and *Clostridioides difficile*—were used to assess antibacterial efficacy (Supplementary Tables S2–S6). In our preliminary tests, **3** was found to exhibit no antimicrobial activity (data not shown). Therefore, **2**, which is structurally similar to **3**, was also expected to lack antibacterial activity. Thus, we evaluated **1** further, comparing it with four commonly used antibiotics [vancomycin (VAN), fidaxomicin (FDX), tigecycline (TGC), and mupirocin (MUP)] and structurally similar phenazine-1-carboxylic acid (PCA) as a control. We determined the lowest minimum inhibitory concentration at which at least 50% of the isolates in a test population are inhibited (MIC_50_) [30] for each bacterial species (Table 3). Remarkably, overall **1** showed relatively high antibacterial activity (0.06–0.12 mg L^−1^), while PCA showed no activity at the concentration employed (8 mg L^−1^). Given its relatively low cytotoxicity and potent antibacterial effects, **1** is a promising drug candidate for the oral or injectable treatment of various infections. If any issues regarding the metabolism or excretion of **1** arise, it may alternatively be evaluated as a candidate topical drug. Comparing the antibacterial activity with the structurally similar dehydrophenazines, griseolutein B from *Streptomyces griseoluteus* P-37 was reported to possess an MIC_50_ of 0.2–0.4 µg mL^−1^ against Gram-positive *Streptococcus pyogenes* and *Bacillus anthracis* [39]. Additionally, compared with the 5,10-dehydrophenazines with the *N*-hydroxyacetyl group from *Kitasatospora* sp. HKI714 reported by Heine et al. showed a high MIC_50_ (100 µg mL^−1^) comparable to that of PCA for methicillin-resistant *S. aureus* SG 511 [40]. These results suggest that additional functional groups (6-hydroxymethyl and/or 9-methoxy groups) in **1** may play important roles in the antibacterial activity.

**Table 3:** MIC_50_ of griseolutein T (**1**) and known antibiotics against clinical isolates

	MIC_50_ (mg L^−1^)					
	1	PCA	VAN	FDX	TGC	MUP
MRSA	0.12	>8	1	4	1	0.25
MSSA	0.12	>8	1	8	0.25	0.25
VRE	0.12	>8	16	2	0.06	0.5
*C. difficile*	0.06	8	0.25	<0.016	<0.016	>8

*MIC*_50_ the lowest minimum inhibitory concentration at which at least 50% of the isolates in a test population are inhibited [44], *MRSA* methicillin-resistant *Staphylococcus aureus*, *MSSA* methicillin-susceptible *Staphylococcus aureus*, *VRE* vancomycin-resistant enterococci, *C. difficileClostridioides difficile*, *PCA* phenazine-1-carboxylic acid (tubermycin B), *VAN* vancomycin, *FDX* fidaxomicin, *TGC* tigecycline, and *MUP* mupirocin

Notably, **1** exhibited higher growth inhibition activity (0.12 mg L^−1^) than commercially available antibiotics, such as VAN, TGC, and MUP, against both MSSA and MRSA, which are significant pathogens in bloodstream infections and opportunistic epidermal infections [41]. *C. difficile* causes severe enteric infections, and FDX is the main antibiotic used for treatment. VRE is a common cause of bloodstream and surgical site infections, and it is noteworthy that **1** exhibited greater efficacy against VRE than VAN, FDX, and MUP. Although VAN remains an effective agent against multidrug-resistant MRSA and *C. difficile* infections, the emergence of diverse VRE strains worldwide poses a serious threat to human health [42]. Since **1** surpasses VAN in antibacterial potency against both MRSA and *C. difficile*, it may offer a promising novel therapeutic option. FDX is a well-established drug to treat infections caused by *C. difficile* [43]. In comparison to FDX, **1** exhibited slightly weaker antibacterial activity against *C. difficile*, but the MIC_50_ of **1** indicates sufficient clinical efficacy. In cases of co-infection with multiple pathogens, a combination of antimicrobial agents may be preferable to using **1** alone. Similar to TGC, **1** exhibited broad efficacy against multidrug-resistant pathogens, albeit with species-specific variations. While TGC is administered intravenously, its use in bacteremia is limited because of its extensive tissue distribution and low blood concentration [44]. Future investigations should explore whether the distribution of **1** is selective and organ-specific, potentially positioning it as an alternative to TGC for the treatment of bacteremia.

## Supplementary information

Supplementary material

## Data Availability

All data generated or analyzed during this study are included in this published article and its Supplementary Information files, which are available at *the Journal of Antibiotics* website.

## References

[1] Hutchings MI, Truman AW, Wilkinson B. Antibiotics: past, present and future. Curr Opin Microbiol. 2019;51:72–80. 31733401 10.1016/j.mib.2019.10.008

[2] Belknap KC, Park CJ, Barth BM, Andam CP. Genome mining of biosynthetic and chemotherapeutic gene clusters in *Streptomyces* bacteria. Sci Rep. 2020;10:2003. 32029878 10.1038/s41598-020-58904-9 PMC7005152

[3] Navarro-Munoz JC, Selem-Mojica N, Mullowney MW, Kautsar SA, Tryon JH, Parkinson EI, et al. A computational framework to explore large-scale biosynthetic diversity. Nat Chem Biol. 2020;16:60–8. 31768033 10.1038/s41589-019-0400-9 PMC6917865

[4] van der Heul HU, Bilyk BL, McDowall KJ, Seipke RF, van Wezel GP. Regulation of antibiotic production in Actinobacteria: new perspectives from the post-genomic era. Nat Prod Rep. 2018;35:575–604. 29721572 10.1039/c8np00012c

[5] Onaka H, Mori Y, Igarashi Y, Furumai T. Mycolic acid-containing bacteria induce natural-product biosynthesis in *Streptomyces* species. Appl Environ Microbiol. 2011;77:400–6. 21097597 10.1128/AEM.01337-10 PMC3020563

[6] Traxler MF, Watrous JD, Alexandrov T, Dorrestein PC, Kolter R. Interspecies interactions stimulate diversification of the *Streptomyces coelicolor* secreted metabolome. mBio. 2013;4:e00459–13. 10.1128/mBio.00459-13 PMC3747584 23963177

[7] Derewacz DK, Covington BC, McLean JA, Bachmann BO. Mapping microbial response metabolomes for induced natural product discovery. ACS Chem Biol. 2015;10:1998–2006. 26039241 10.1021/acschembio.5b00001 PMC4987304

[8] Adnani N, Chevrette MG, Adibhatla SN, Zhang F, Yu Q, Braun DR, et al. Coculture of marine invertebrate-associated bacteria and interdisciplinary technologies enable biosynthesis and discovery of a new antibiotic, keyicin. ACS Chem Biol. 2017;12:3093–102. 29121465 10.1021/acschembio.7b00688 PMC5973552

[9] Lee N, Kim W, Chung J, Lee Y, Cho S, Jang KS, et al. Iron competition triggers antibiotic biosynthesis in *Streptomyces coelicolor* during coculture with *Myxococcus xanthus*. ISME J. 2020;14:1111–24. 31992858 10.1038/s41396-020-0594-6 PMC7174319

[10] Hoshino S, Onaka H, Abe I. Activation of silent biosynthetic pathways and discovery of novel secondary metabolites in actinomycetes by co-culture with mycolic acid-containing bacteria. J Ind Microbiol Biotechnol. 2019;46:363–74. 30488365 10.1007/s10295-018-2100-y

[11] Asamizu S, Pramana AAC, Kawai SJ, Arakawa Y, Onaka H. Comparative metabolomics reveals a bifunctional antibacterial conjugate from combined-culture of *Streptomyces hygroscopicus* HOK021 and *Tsukamurella pulmonis* TP-B0596. ACS Chem Biol. 2022;17:2664–72. 36074093 10.1021/acschembio.2c00585

[12] Hagihara R, Katsuyama Y, Sugai Y, Onaka H, Ohnishi Y. Novel desferrioxamine derivatives synthesized using the secondary metabolism-specific nitrous acid biosynthetic pathway in *Streptomyces davawensis*. J Antibiot. 2018;71:911–9. 10.1038/s41429-018-0088-1 30120394

[13] Ozaki T, Sugiyama R, Shimomura M, Nishimura S, Asamizu S, Katsuyama Y, et al. Identification of the common biosynthetic gene cluster for both antimicrobial streptoaminals and antifungal 5-alkyl-1,2,3,4-tetrahydroquinolines. Org Biomol Chem. 2019;17:2370–8. 30629078 10.1039/c8ob02846j

[14] Hoshino S, Okada M, Wakimoto T, Zhang H, Hayashi F, Onaka H, et al. Niizalactams A-C, multicyclic macrolactams isolated from combined culture of *Streptomyces* with mycolic acid-containing bacterium. J Nat Prod. 2015;78:3011–7. 26624939 10.1021/acs.jnatprod.5b00804

[15] Hoshino S, Okada M, Awakawa T, Asamizu S, Onaka H, Abe I. Mycolic acid containing bacterium stimulates tandem cyclization of polyene macrolactam in a lake sediment derived rare actinomycete. Org Lett. 2017;19:4992–5. 28880091 10.1021/acs.orglett.7b02508

[16] Hoshino S, Ozeki M, Wong CP, Zhang H, Hayashi F, Awakawa T, et al. Mirilactams C-E, novel polycyclic macrolactams isolated from combined-culture of *Actinosynnema mirum* NBRC 14064 and mycolic acid-containing bacterium. Chem Pharm Bull. 2018;66:660–7. 10.1248/cpb.c18-00143 29863068

[17] Asamizu S, Ozaki T, Teramoto K, Satoh K, Onaka H. Killing of mycolic acid-containing bacteria aborted induction of antibiotic production by *Streptomyces* in combined-culture. PLoS ONE. 2015;10:e0142372. 26544713 10.1371/journal.pone.0142372 PMC4636228

[18] Yanagisawa M, Asamizu S, Satoh K, Oono Y, Onaka H. Effects of carbon ion beam-induced mutagenesis for the screening of RED production-deficient mutants of *Streptomyces coelicolor* JCM4020. PLoS ONE. 2022;17:e0270379. 35834474 10.1371/journal.pone.0270379 PMC9282665

[19] Lei YK, Asamizu S, Ishizuka T, Onaka H. Regulation of multidrug efflux pumps by TetR family transcriptional repressor negatively affects secondary metabolism in *Streptomyces**coelicolor* A3(2). Appl Environ Microb. 2023;89:e0182222. 10.1128/aem.01822-22 PMC10056966 36790176

[20] Lei Y, Onaka H, Asamizu S. Transcriptionally induced nucleoid-associated protein-like *ccr1* in combined-culture serves as a global effector of *Streptomyces* secondary metabolism. Front Microbiol. 2024;15:1422977. 39070263 10.3389/fmicb.2024.1422977 PMC11272600

[21] O’Neill J. Tackling drug-resistant infections globally: final report and recommendations. Rev Antimicrob Resist. London, United Kingdom: Wellcome Trust; 2016; p. 1–80.

[22] WHO global action plan on antimicrobial resistance. Geneva, Switzerland: World Health Organization; 2016.

[23] Kato M, Asamizu S, Onaka H. Intimate relationships among actinomycetes and mycolic acid-containing bacteria. Sci Rep. 2022;12:7222. 35508597 10.1038/s41598-022-11406-2 PMC9068768

[24] Sugiyama R, Nishimura S, Ozaki T, Asamizu S, Onaka H, Kakeya H. 5-Alkyl-1,2,3,4-tetrahydroquinolines, new membrane-interacting lipophilic metabolites produced by combined culture of *Streptomyces nigrescens* and *Tsukamurella pulmonis*. Org Lett. 2015;17:1918–21. 25826296 10.1021/acs.orglett.5b00607

[25] Sugiyama R, Nishimura S, Ozaki T, Asamizu S, Onaka H, Kakeya H. Discovery and total synthesis of streptoaminals: antimicrobial [5,5]-spirohemiaminals from the combined-culture of *Streptomyces nigrescens* and *Tsukamurella pulmonis*. Angew Chem Int Ed Engl. 2016;55:10278–82. 27459894 10.1002/anie.201604126

[26] Kusumoto M, Ogura Y, Gotoh Y, Iwata T, Hayashi T, Akiba M. Colistin-resistant mcr-1-positive pathogenic *Escherichia coli* in Swine, Japan, 2007-2014. Emerg Infect Dis. 2016;22:1315–7. 27314277 10.3201/eid2207.160234 PMC4918142

[27] Nakamura S. Structure of griseolutein B. J Antibiot. 1959;12:26–7. 13641132

[28] Yagishita K. Production of phenazine compounds by *Stretomyces griseoluteus*. J Antibiot Ser A. 1960;13:83–96.

[29] Nakamura S, Wang EL, Murase M, Maeda K, Umezawa H. Structure of griseolutein A. J Antibiot 1959;12:55–8. 13654145

[30] CLSI. Performance standards for antimicrobial susceptibility testing. 26th Edition. Wayne, PA: Clinical and Laboratory Standards Institute; 2016.

[31] Charbon G, Klitgaard RN, Liboriussen CD, Thulstrup PW, Maffioli SI, Donadio S, et al. Iron chelation increases the tolerance of *Escherichia coli* to hyper-replication stress. Sci Rep. 2018;8:10550. 30002429 10.1038/s41598-018-28841-9 PMC6043582

[32] Nakamura S. Studies on structure of griseolutein-B, a *Streptomyces* antibiotic. III. The complete structure. Chem Pharm Bull. 1958;6:547–50.

[33] Nakamura S, Maeda K, Umezawa H. The structure of griseolutein B. J Antibiot. 1964;17:33–6. 14109823

[34] Challand SR, Herbert RB, Holliman FG. A new phenazine synthesis. The synthesis of griseoluteic acid, griseolutein A, and methyl diacetylgriseolutein B. J Chem Soc D Chem Commun. 1970:1423–5.

[35] Price-Whelan A, Dietrich LE, Newman DK. Rethinking ‘secondary’ metabolism: physiological roles for phenazine antibiotics. Nat Chem Biol. 2006;2:71–8. 16421586 10.1038/nchembio764

[36] Vind K, Maffioli S, Fernandez Ciruelos B, Waschulin V, Brunati C, Simone M, et al. N-acetyl-cysteinylated streptophenazines from *Streptomyces*. J Nat Prod. 2022;85:1239–47. 35422124 10.1021/acs.jnatprod.1c01123 PMC9150181

[37] Newton GL, Buchmeier N, Fahey RC. Biosynthesis and functions of mycothiol, the unique protective thiol of Actinobacteria. Microbiol Mol Biol Rev. 2008;72:471–94. 18772286 10.1128/MMBR.00008-08 PMC2546866

[38] Nakamura S. Studies on structure of Griseolutein B, a *Streptomyces* antibiotic. I. Characterization and degradation. Chem. Pharm. Bull. 1958;6:539–43.

[39] Ogata Y. Biological studies on the antibiotics produced by *Streptomyces griseoluteus*. II. The effect of griseolutein B in vitro and in vivo. Jpn J Med Sci Biol. 1953;6:493–501. 13142762 10.7883/yoken1952.6.493

[40] Heine D, Martin K, Hertweck C. Genomics-guided discovery of endophenazines from *Kitasatospora* sp. HKI 714. J Nat Prod. 2014;77:1083–7. 24617951 10.1021/np400915p

[41] Tong SY, Davis JS, Eichenberger E, Holland TL, Fowler VG Jr. *Staphylococcus aureus* infections: epidemiology, pathophysiology, clinical manifestations, and management. Clin Microbiol Rev. 2015;28:603–61. 26016486 10.1128/CMR.00134-14 PMC4451395

[42] Ahmed MO, Baptiste KE. Vancomycin-resistant enterococci: a review of antimicrobial resistance mechanisms and perspectives of human and animal health. Microb Drug Resist. 2018;24:590–606. 29058560 10.1089/mdr.2017.0147

[43] Johnson AP, Wilcox MH. Fidaxomicin: a new option for the treatment of *Clostridium difficile* infection. J Antimicrob Chemother. 2012;67:2788–92. 22865382 10.1093/jac/dks302

[44] Yaghoubi S, Zekiy AO, Krutova M, Gholami M, Kouhsari E, Sholeh M, et al. Tigecycline antibacterial activity, clinical effectiveness, and mechanisms and epidemiology of resistance: narrative review. Eur J Clin Microbiol Infect Dis. 2022;41:1003–22. 33403565 10.1007/s10096-020-04121-1 PMC7785128

