# Supplementary material for: Griseolutein T from Streptomyces seoulensis, newly identified via combined-culture with Tsukamurella pulmonis, as an efficacious therapeutic agent against multidrug-resistant bacteria
Source: J Antibiot (Tokyo). 2025 Jul 9;78(9):542–51. doi: 10.1038/s41429-025-00846-3 (PMC12380601; doi:10.1038/s41429-025-00846-3)
Supplement: Supplementary file 1 — Supplementary material [file 41429_2025_846_MOESM1_ESM.docx]

**SUPPLEMENTAL MATERIAL**

**Title:**

Griseolutein T from *Streptomyces seoulensis*, Newly Identified via Combined-culture with *Tsukamurella pulmonis*, as an Efficacious Therapeutic Agent against Multidrug-resistant Bacteria

**Authors:**

Sung-Jin Kawai^1#^, Shumpei Asamizu^2,3,6#^*, Hiroaki Suzuki^1^, Hiroyasu Onaka^2,3,7^, Yoshichika Arakawa^4,8^, Kouji Kimura^4^, and Makoto Ojika^5^

**Affiliations:**

^1^ New Field Pioneering Division, Toyota Boshoku Corporation, 1-1 Toyoda, Kariya, Aichi 448-8651, Japan

^2^ Graduate School of Agricultural and Life Sciences, The University of Tokyo, 1-1-1 Yayoi Bunkyo, Tokyo 113-0033, Japan

^3^ Collaborative Research Institute for Innovative Microbiology, The University of Tokyo 1-1-1 Yayoi, Bunkyo, Tokyo 113-8657, Japan

^4^ Graduate School of Medicine, Nagoya University, 65 Tsurumai, Showa, Nagoya 466-8550, Japan

^5^ Graduate School of Bioagricultural Sciences, Nagoya University, Chikusa, Nagoya 464-8601, Japan

^6^ Engineering Biology Research Center, Kobe University. 1-1 Rokkodai, Nada, Kobe 657-8501, Japan

^7^ Department of Life Science, Faculty of Science, Gakushuin University, Mejiro 1-5-1, Toshima, Tokyo 171-8588, Japan

^8^ Present address: Department of Bacteriology, Fujita Health University School of Medicine, Toyoake, Aichi 470-1192, Japan

^#^These authors contributed equally to this study.

***Corresponding author:**

SA: shumpei.asamizu@port.kobe-u.ac.jp

**Contents of supplementary data**

**Supplementary figure S1**

Purification scheme of compounds **1**, **2** and **3** from HEK131 mono-culture.

**Supplementary figure S2**

Purification scheme of compounds **1**, **2** and **3** from HEK131 and *T. pulmonis* combined-culture.

**Supplementary figure S3**

ESI-TOF MS (+)

groseolutein T (**1**), groseolutein C (**2**), griseolutein D (**3**), griseoluteic acid (**4**), griseolutein A (**5**)

**Supplementary figure S4**

^1^H NMR of griseolutein T (**1**) (400 MHz, DMSO-*d*_6_)

**Supplementary figure S5**

^13^C NMR of griseolutein T (**1**) (100 MHz, DMSO-*d*_6_)

**Supplementary figure S6**

COSY of griseolutein T (**1**) (600 MHz, DMSO-*d*_6_)

**Supplementary figure S7**

HSQC of griseolutein T (**1**) (600 MHz, DMSO-*d*_6_)

**Supplementary figure S8**

HMBC of griseolutein T (**1**) (400 MHz, DMSO-*d*_6_)

**Supplementary figure S9**

^1^H NMR of griseolutein C (**2**) (400 MHz, DMSO-*d*_6_)

**Supplementary figure S10**

^13^C NMR of griseolutein C (**2**) (100 MHz, DMSO-*d*_6_)

**Supplementary figure S11**

HSQC of griseolutein C (**2**) (400 MHz, DMSO-*d*_6_)

**Supplementary figure S12**

HMBC of griseolutein C (**2**) (400 MHz, DMSO-*d*_6_)

**Supplementary figure S13**

^1^H NMR of griseolutein D (**3**) (400 MHz, DMSO-*d*_6_)

**Supplementary figure S14**

^13^C NMR of griseolutein D (**3**) (100 MHz, DMSO-*d*_6_)

**Supplementary figure S15**

DQF-COSY of griseolutein D (**3**) (100 MHz, DMSO-*d*_6_)

**Supplementary figure S16**

HSQC of griseolutein D (**3**) (100 MHz, DMSO-*d*_6_)

**Supplementary figure S17**

HMBC of griseolutein D (**3**) (100 MHz, DMSO-*d*_6_)

**Supplementary figure S18**

Comparison of production titers of compounds 1–5

**Supplementary figure S19**

Cytotoxicity test

**Supplementary table S1**

^1^H NMR spectral data of compound **4** in DMSO-*d*_6_

**Supplementary table S2**

List of clinical isolates of MRSA used for antibacterial tests

**Supplementary table S3**

List of clinical isolates of MSSA used for antibacterial tests

**Supplementary table S4**

List of clinical isolates of VRE used for antibacterial tests

**Supplementary table S5**

List of clinical isolates of *C. difficile* used for antibacterial tests

**Supplementary table S6**

List of type strains of MRSA (ATCC43300), MSSA (ATCC29213), VRE (ATCC51299), and *C. difficile* (ATCC700057) used for antibacterial tests

**Supplementary table S7**

Relative ion intensity of isotope peaks for 2 and 3


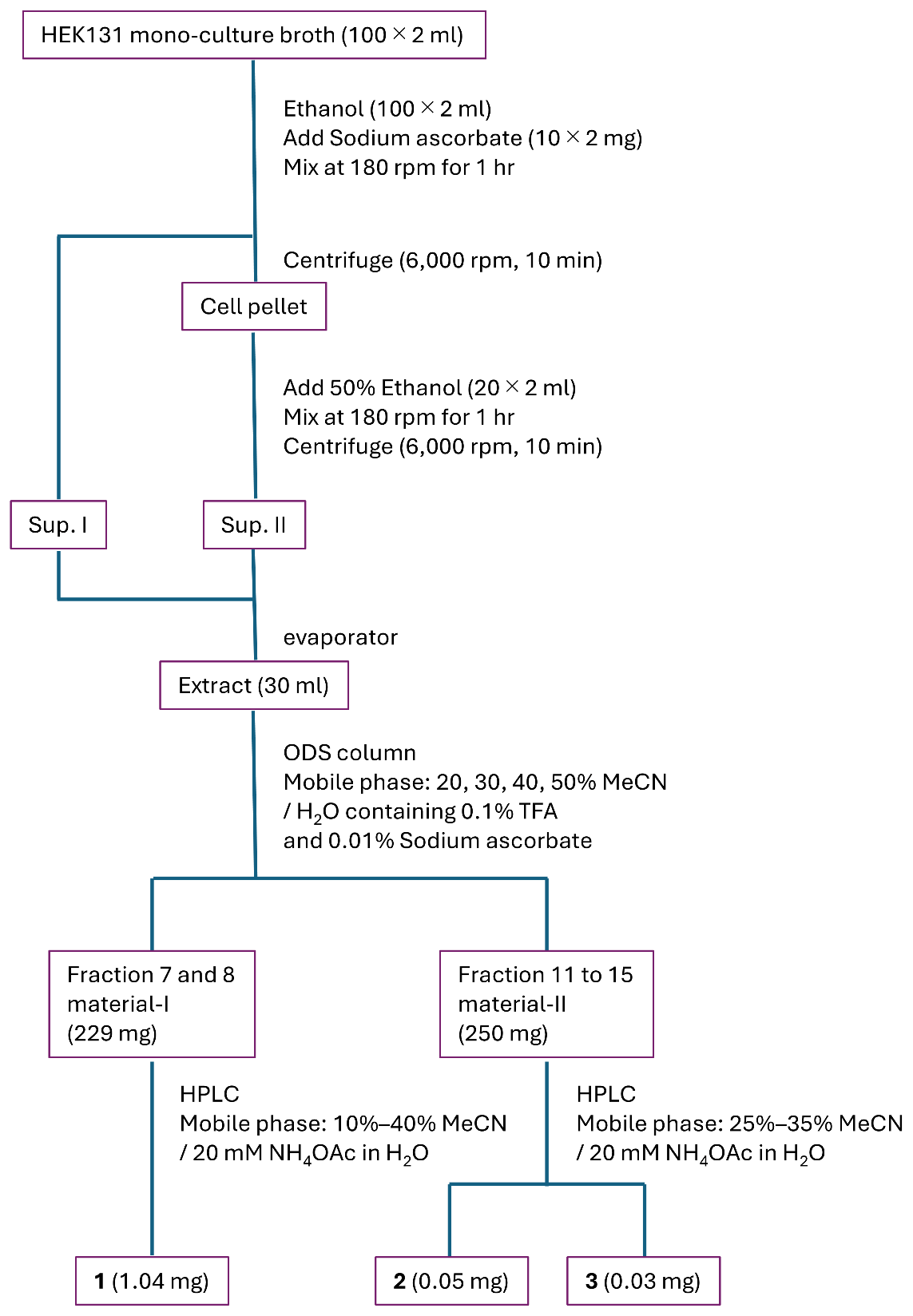


**Supplementary figure S1**

Purification scheme of compounds **1**, **2** and **3** from HEK131 mono-culture.


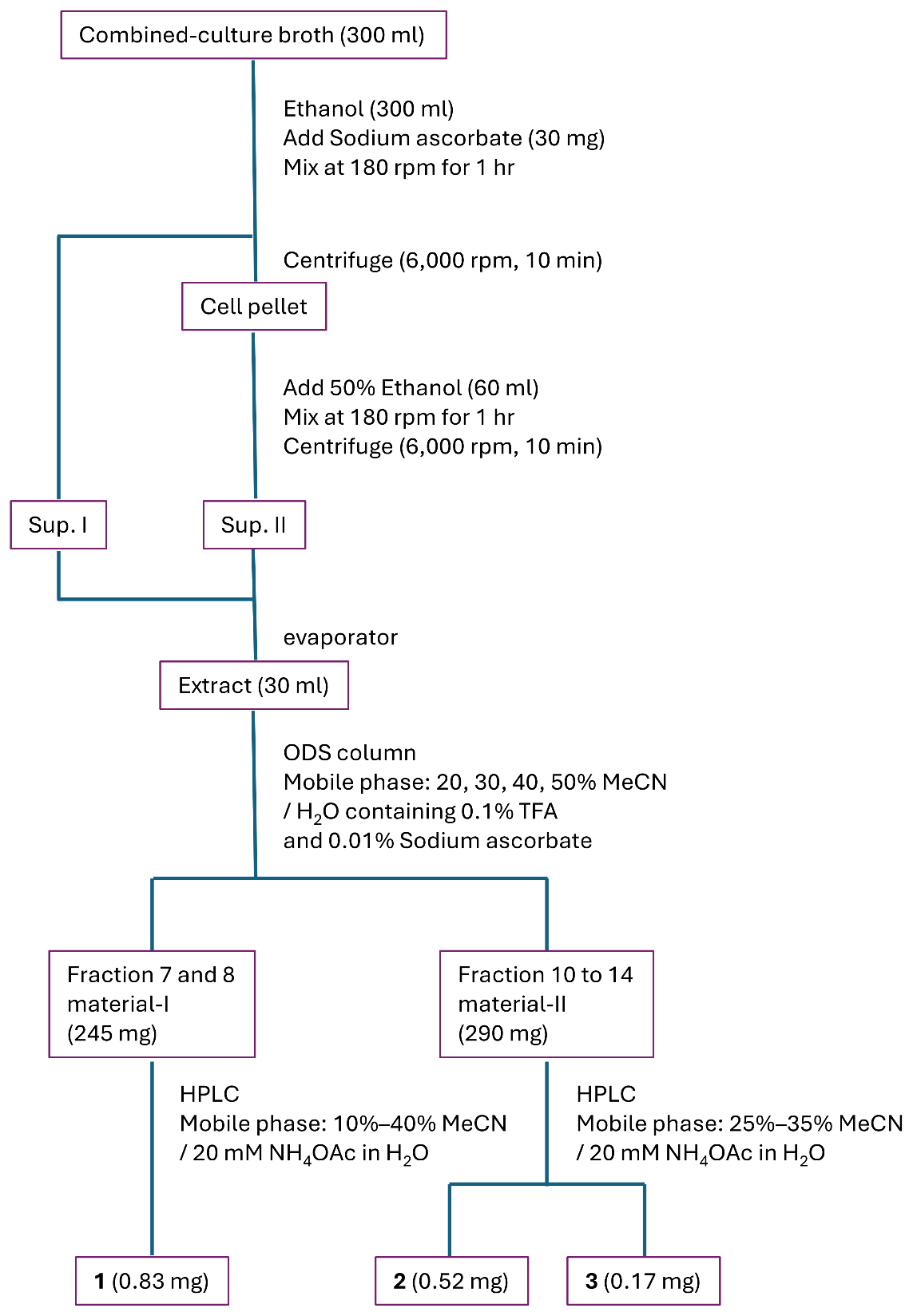


**Supplementary figure S2**

Purification scheme of compounds **1**, **2** and **3** from HEK131 and *T. pulmonis* combined-culture.


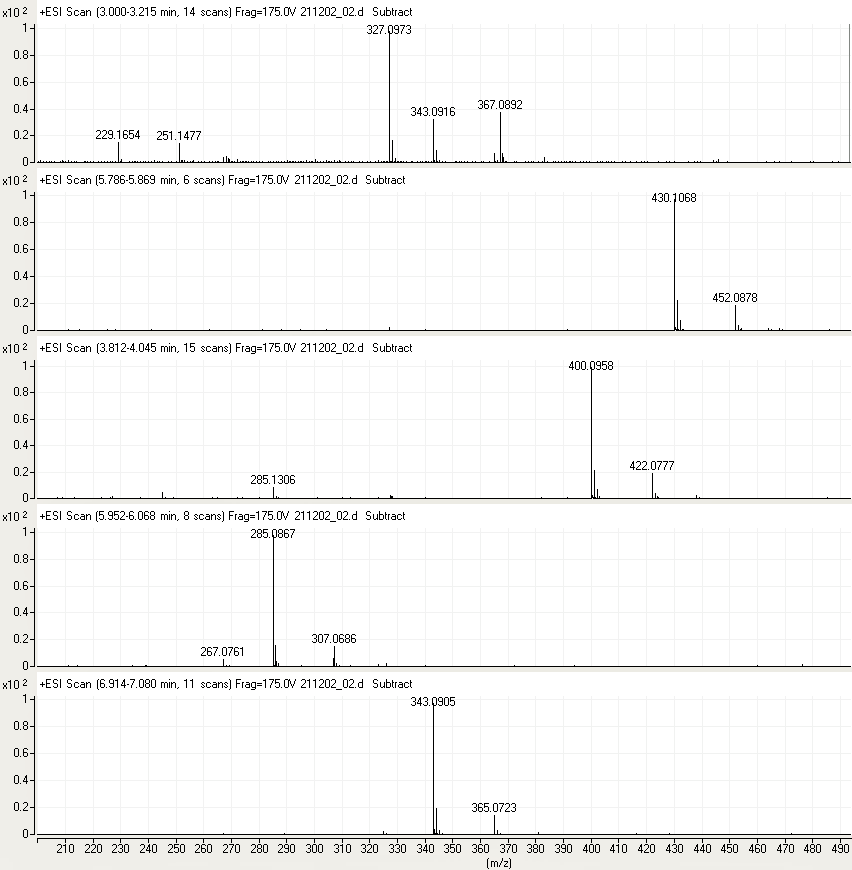
**Supplementary figure S3**

ESI-TOF MS (+)

(from top)

groseolutein T (**1**), groseolutein C (**2**), griseolutein D (**3**), griseoluteic acid (**4**), griseolutein A (**5**)


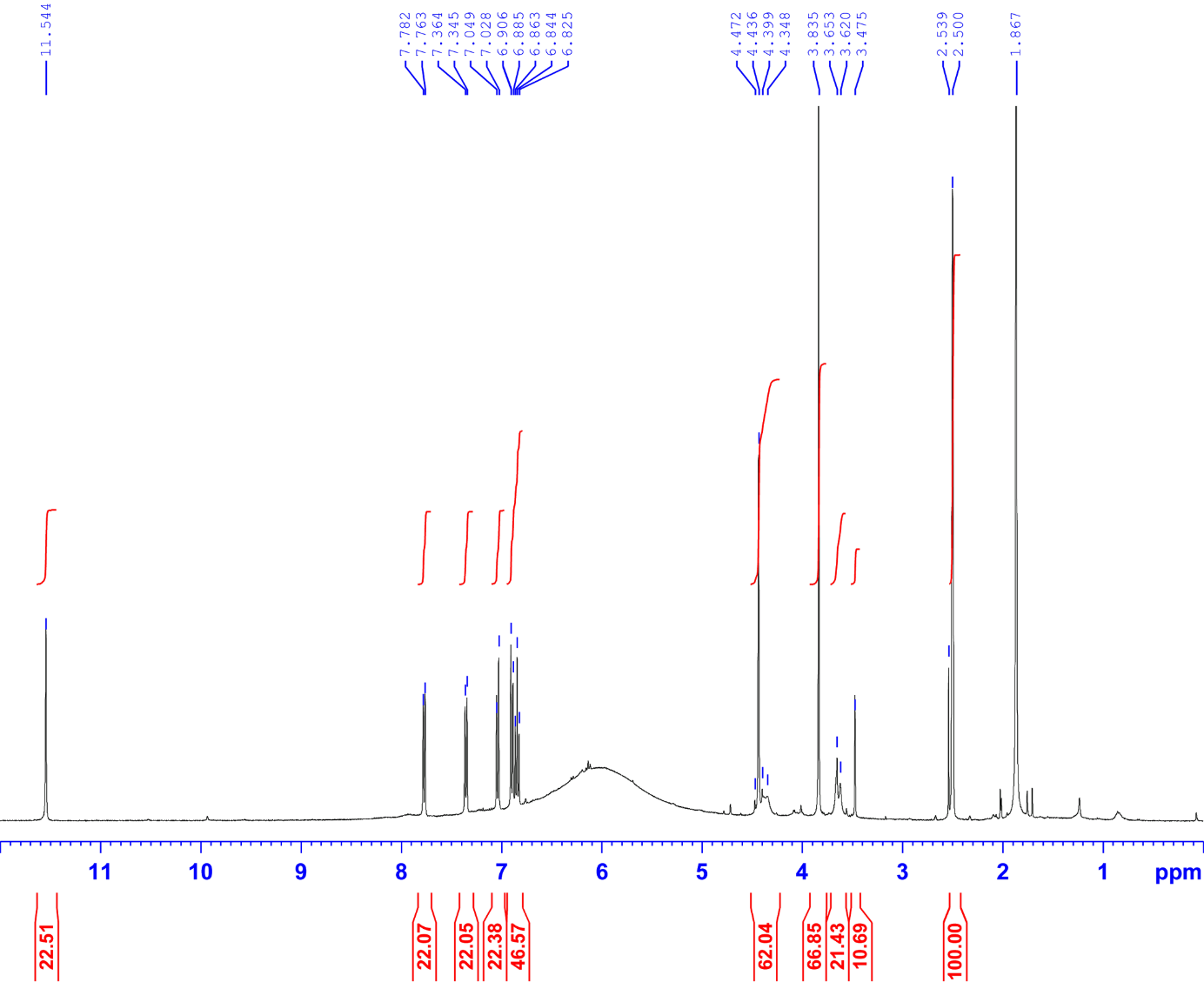
**Supplementary figure S4**

^1^H NMR of griseolutein T (**1**) (400 MHz, DMSO-*d*_6_)


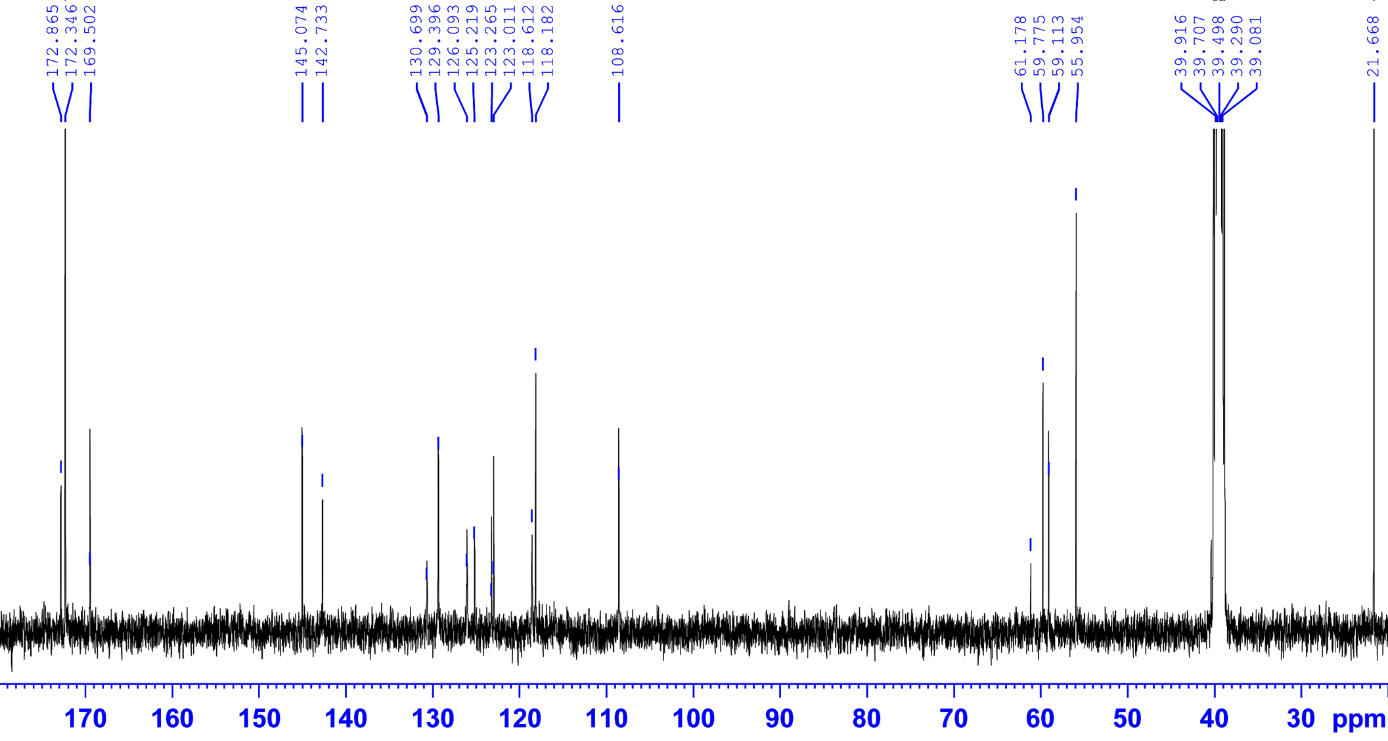
**Supplementary figure S5**

^13^C NMR of griseolutein T (**1**) (100 MHz, DMSO-*d*_6_)


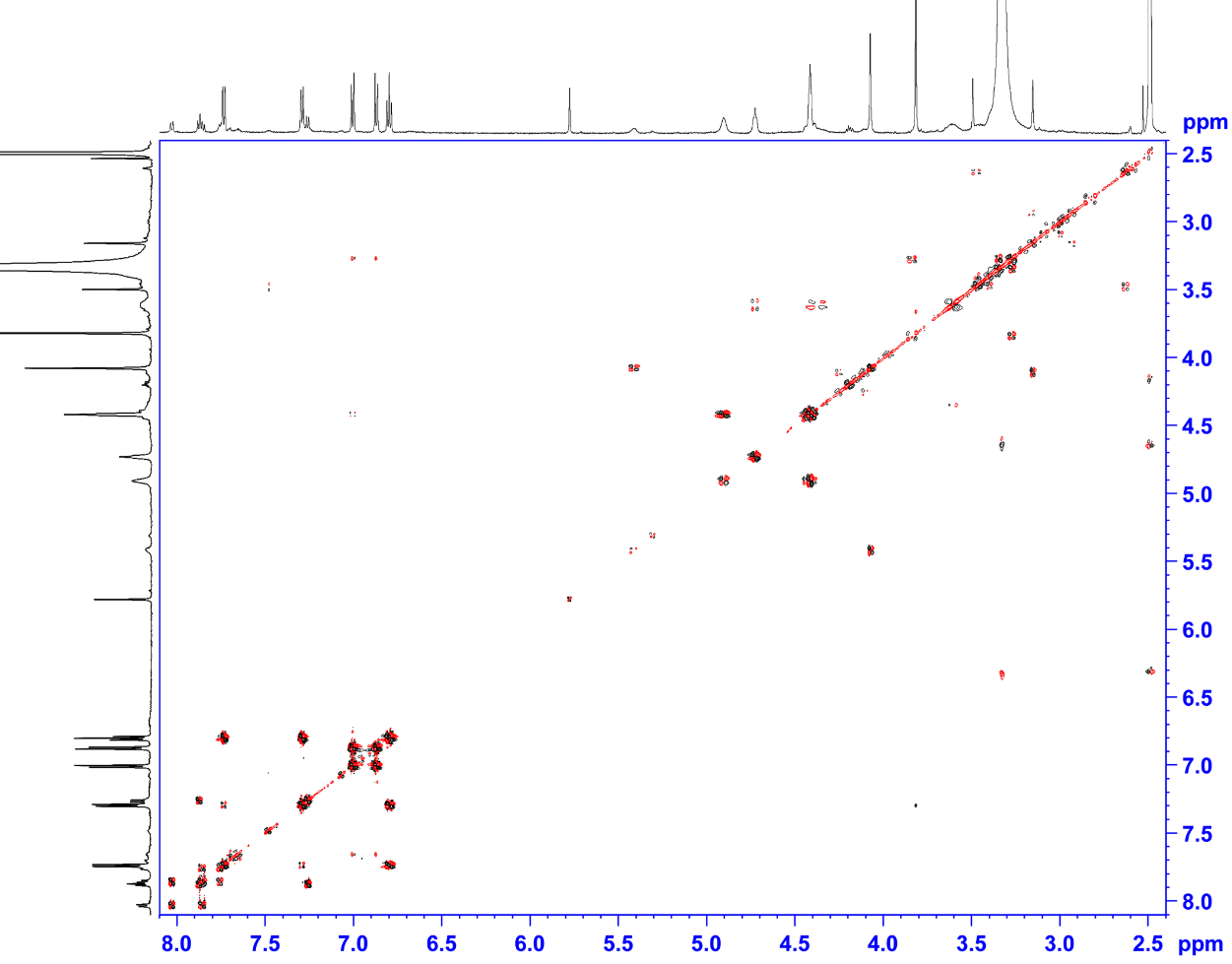
**Supplementary figure S6**

COSY of griseolutein T (**1**) (600 MHz, DMSO-*d*_6_)


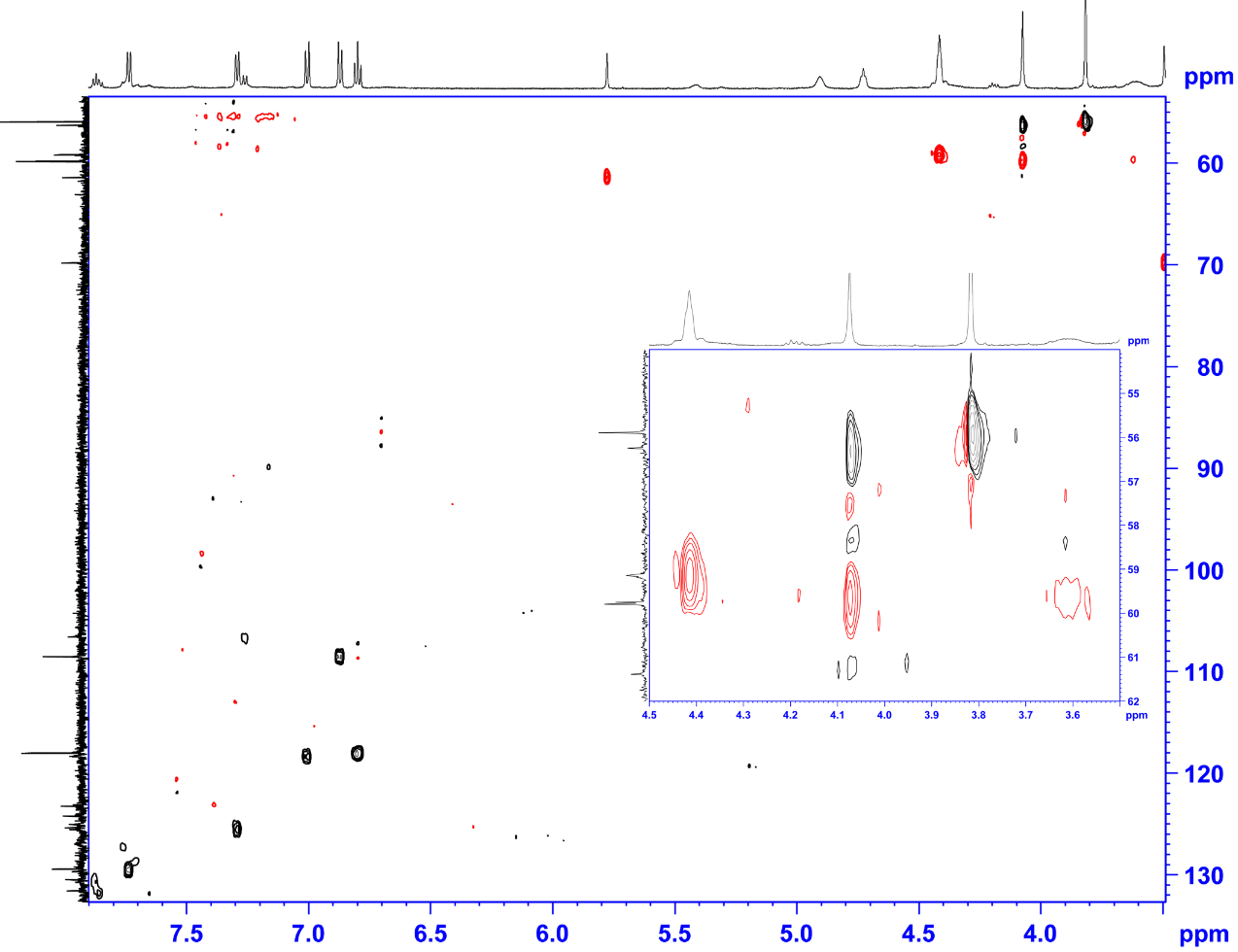


**Supplementary figure S7**

HSQC of griseolutein T (**1**) (600 MHz, DMSO-*d*_6_)


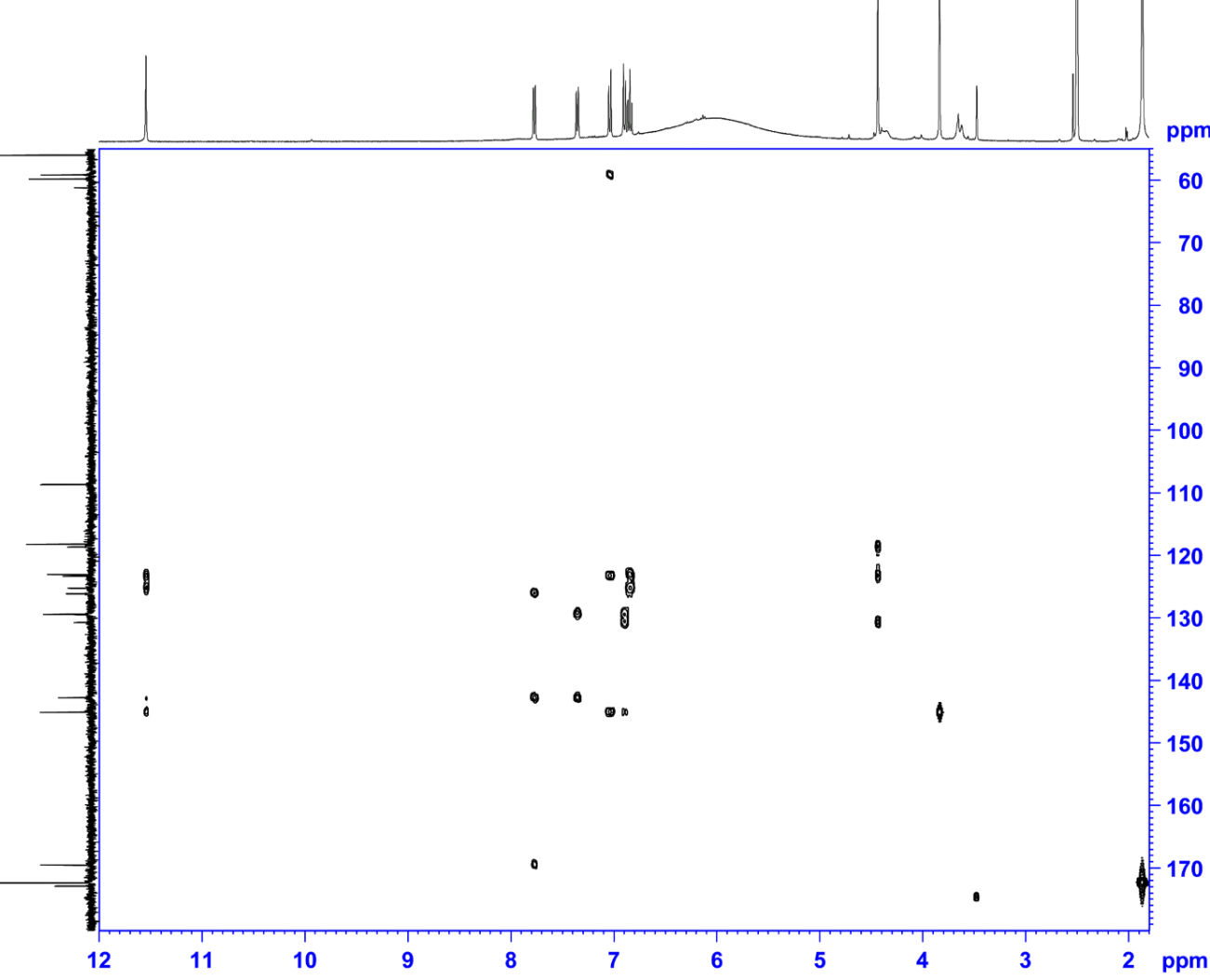
**Supplementary figure S8**

HMBC of griseolutein T (**1**) (400 MHz, DMSO-*d*_6_)


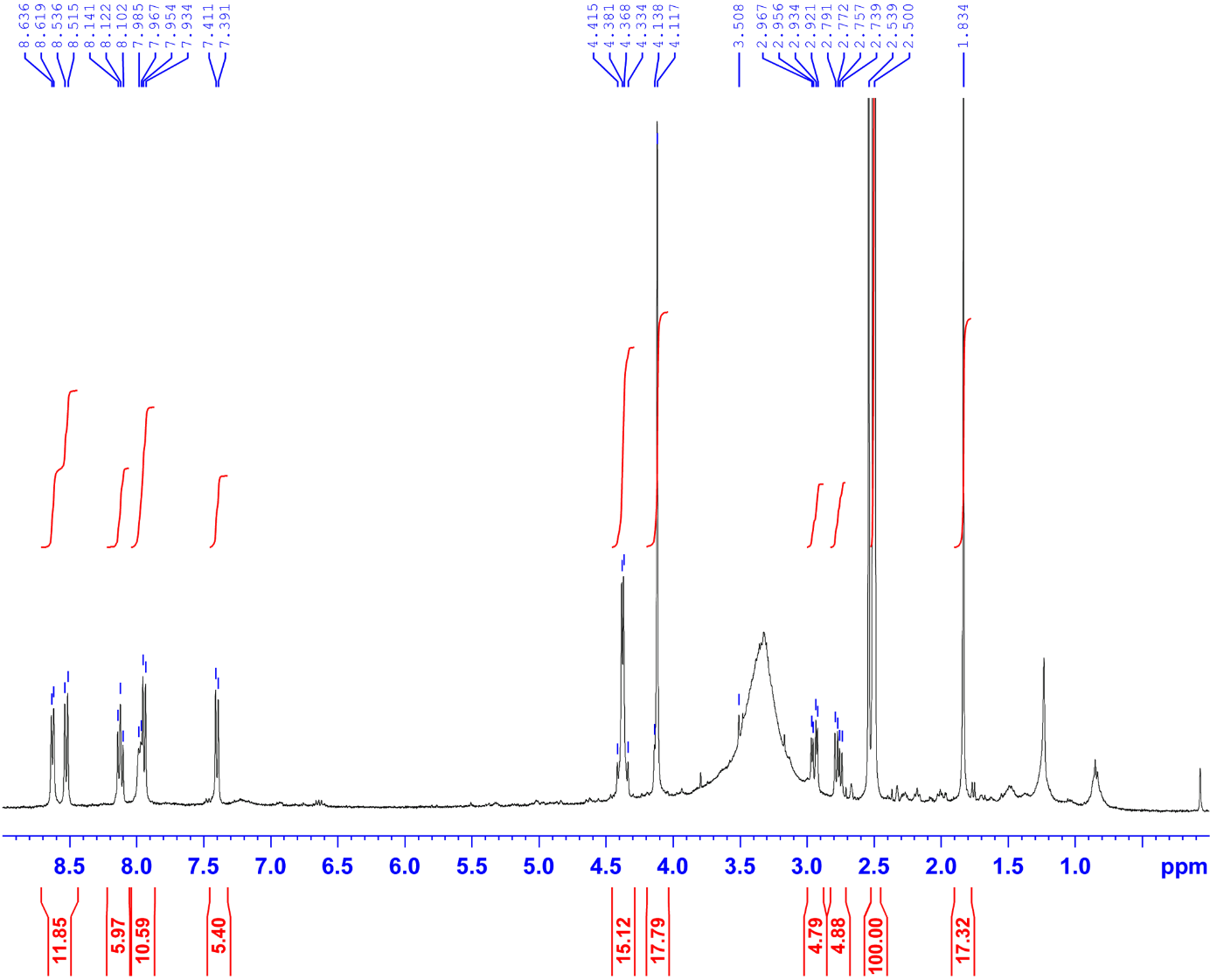
**Supplementary figure S9**

^1^H NMR of griseolutein C (**2**) (400 MHz, DMSO-*d*_6_)


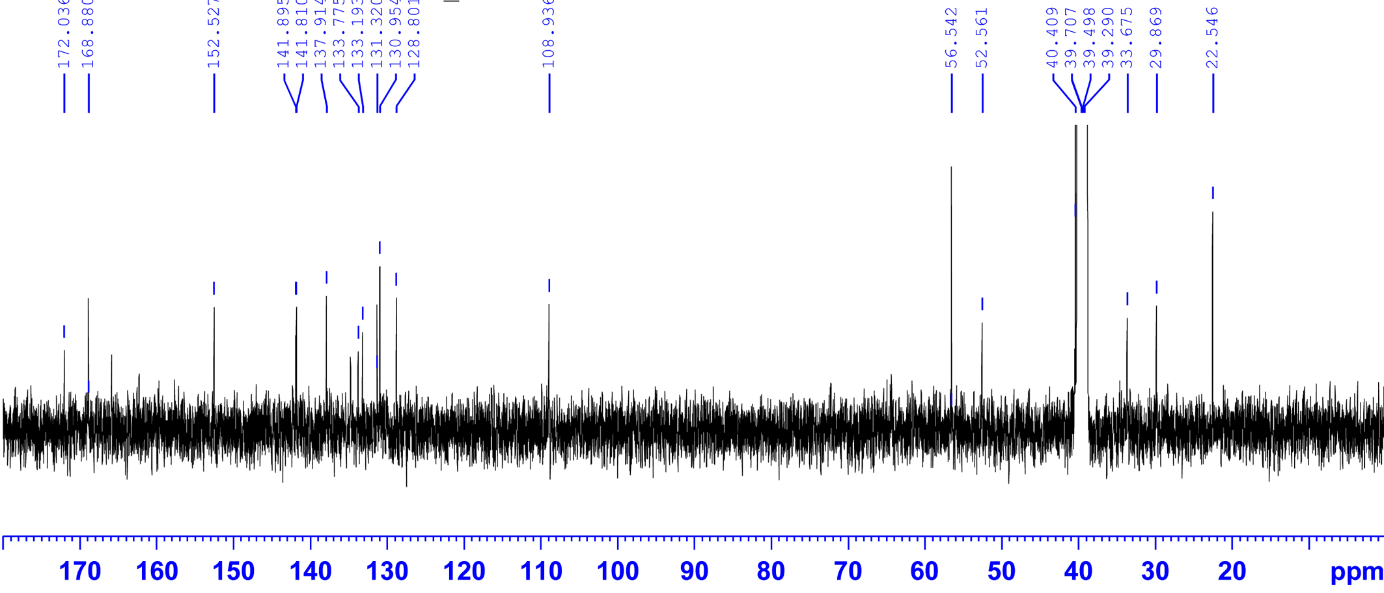
**Supplementary figure S10**

^13^C NMR of griseolutein C (**2**) (100 MHz, DMSO-*d*_6_)


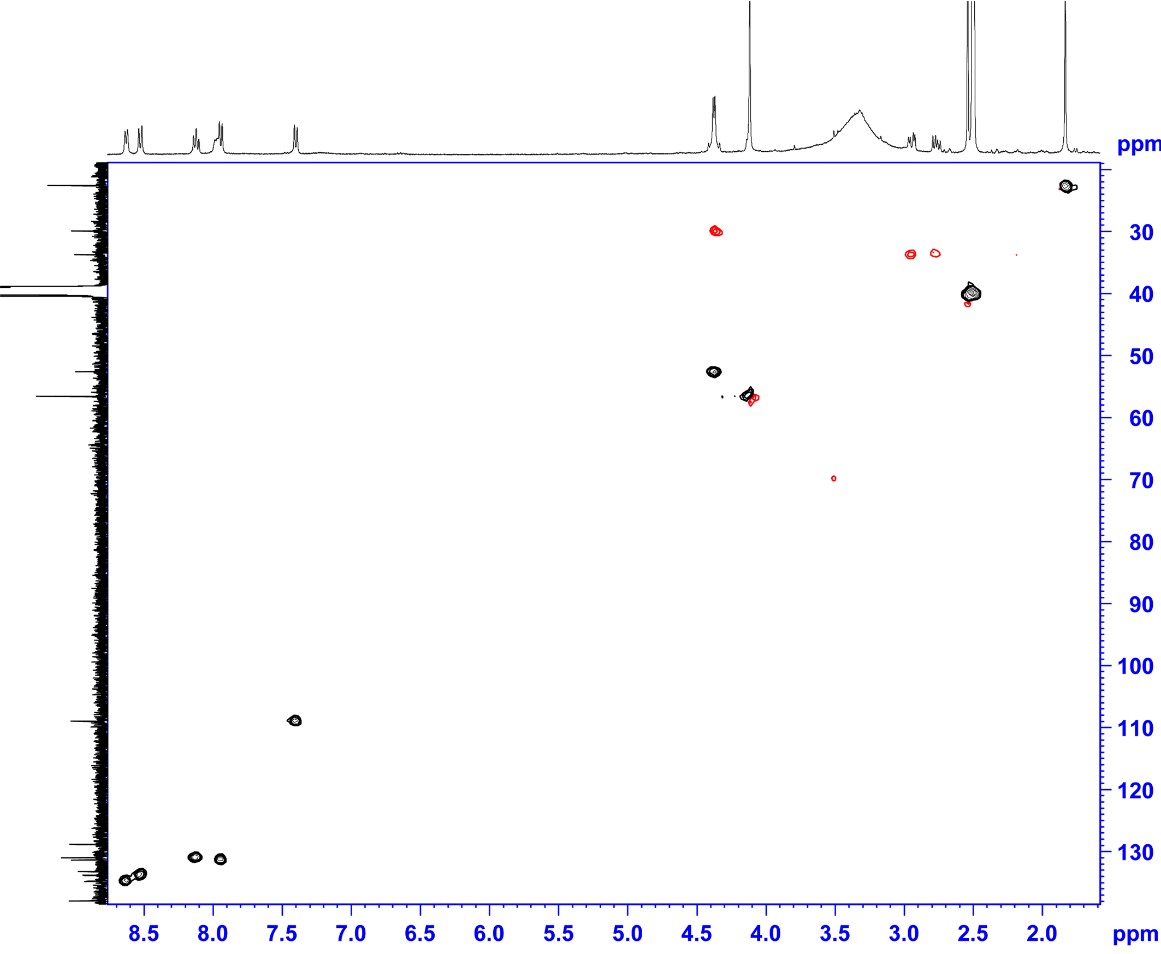
**Supplementary figure S11**

HSQC of griseolutein C (**2**) (400 MHz, DMSO-*d*_6_)


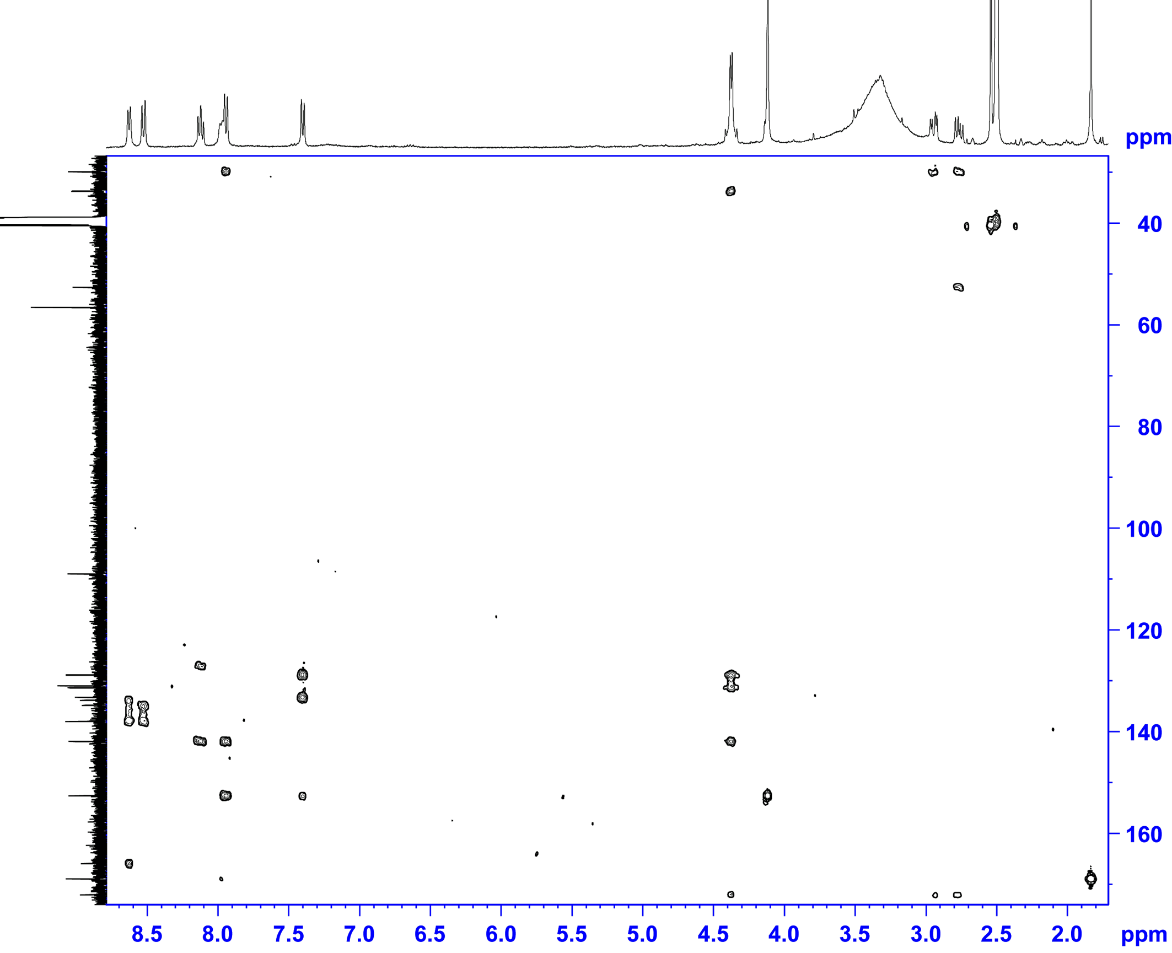
**Supplementary figure S12**

HMBC of griseolutein C (**2**) (400 MHz, DMSO-*d*_6_)


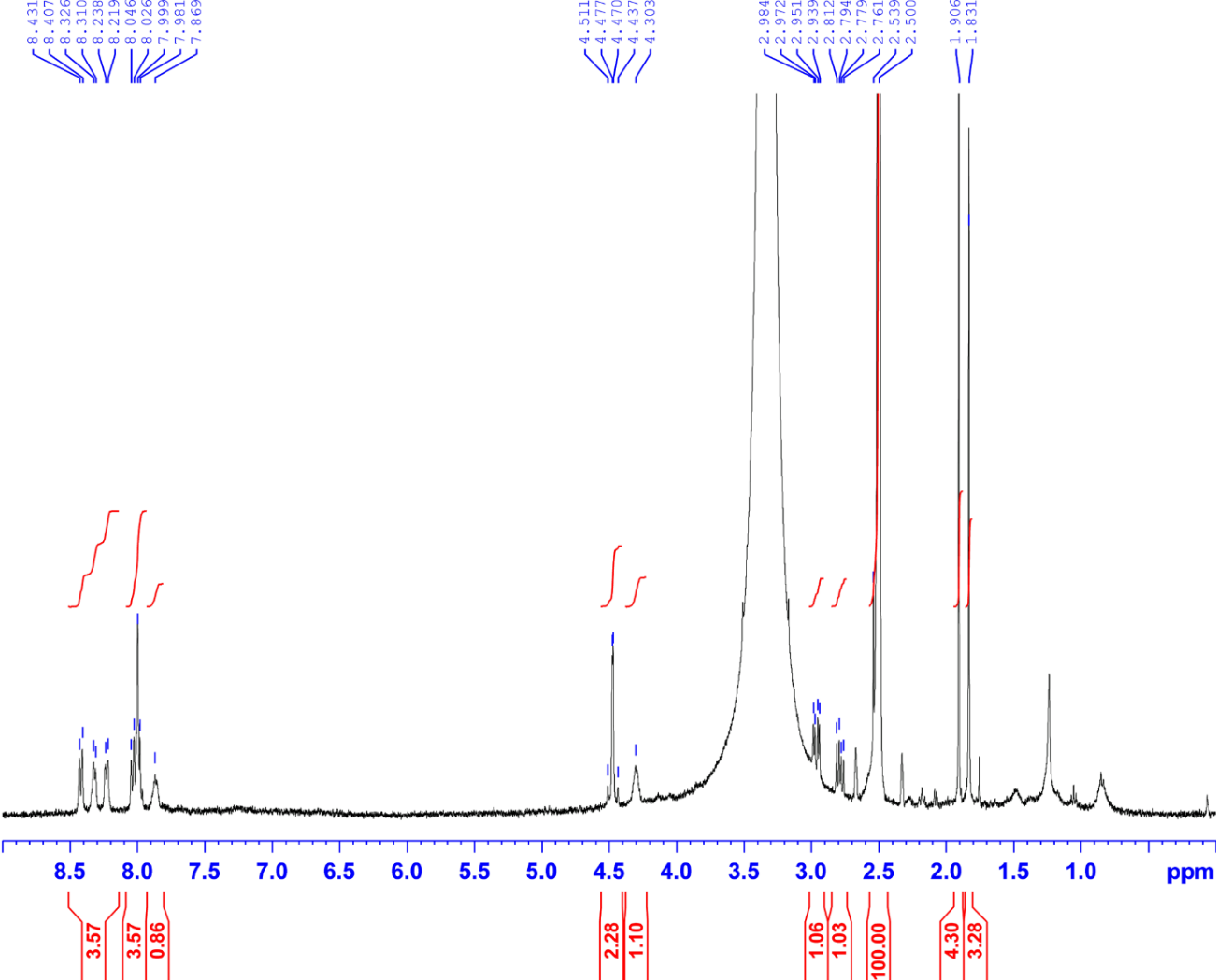
**Supplementary figure S13**

^1^H NMR of griseolutein D (**3**) (400 MHz, DMSO-*d*_6_)


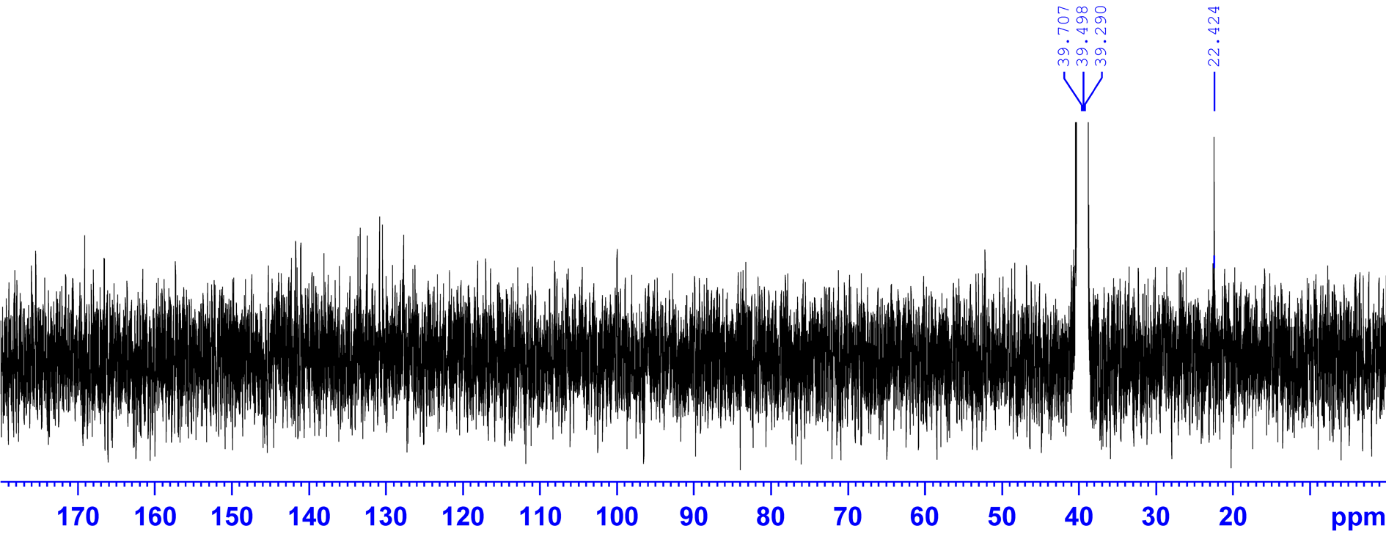
**Supplementary figure S14**

^13^C NMR of griseolutein D (**3**) (100 MHz, DMSO-*d*_6_)


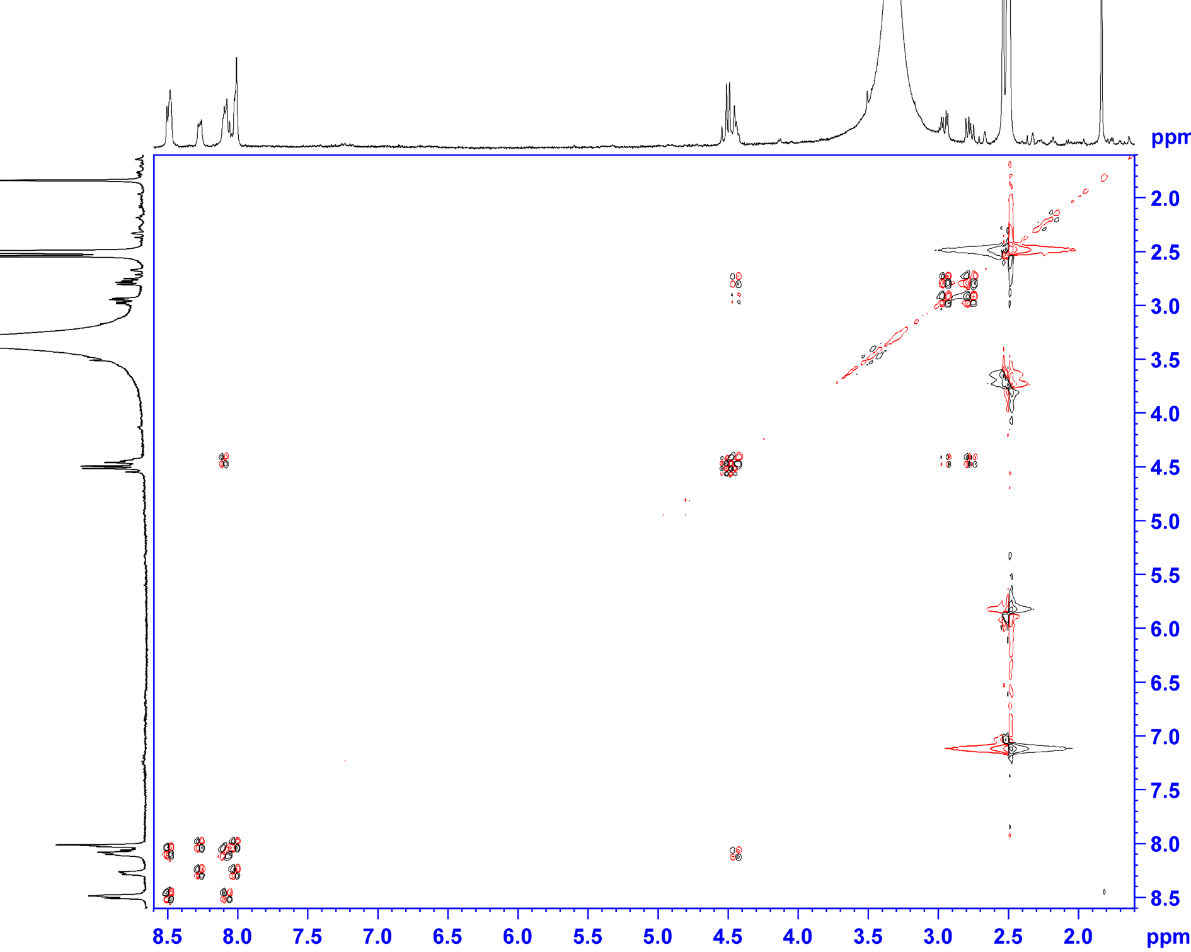
**Supplementary figure S15**

DQF-COSY of griseolutein D (**3**) (100 MHz, DMSO-*d*_6_)


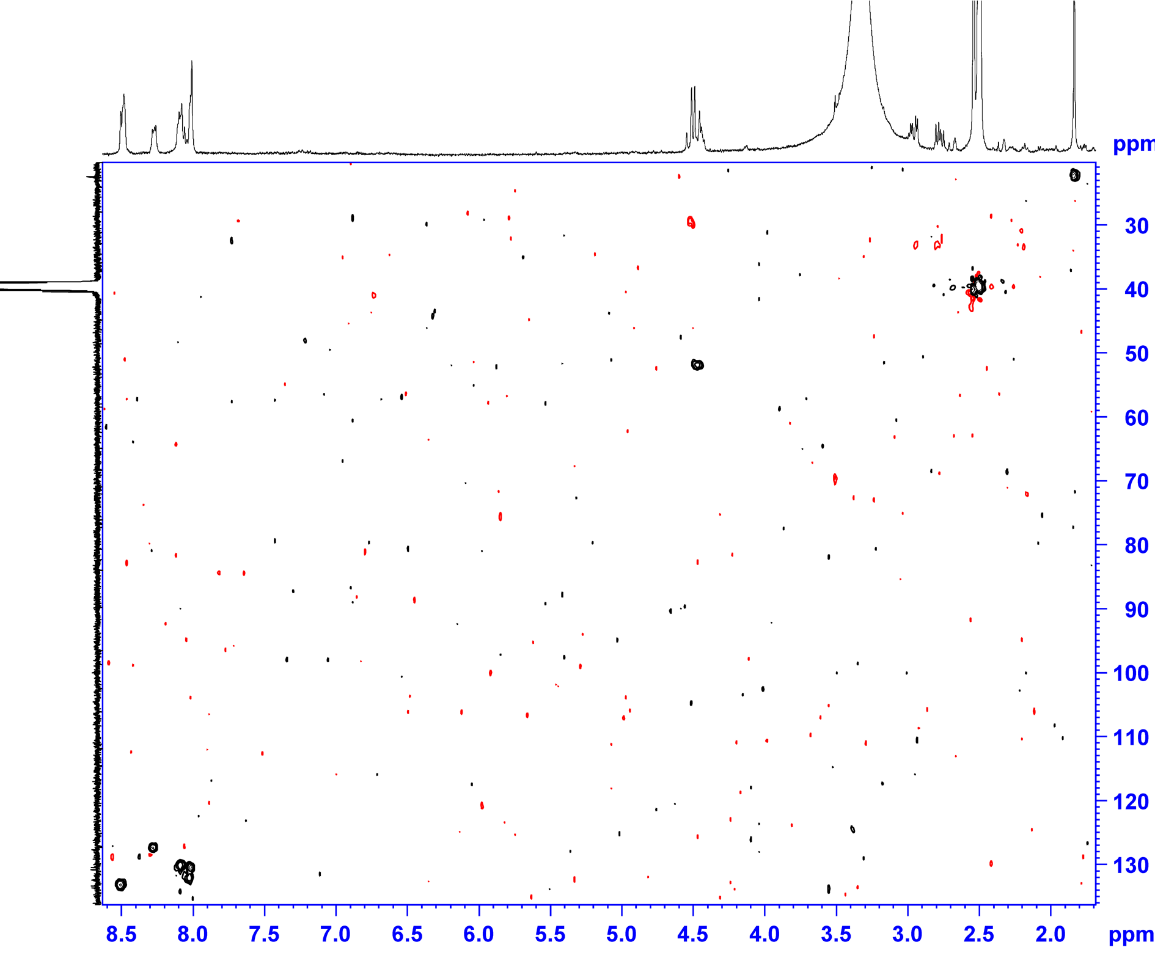
**Supplementary figure S16**

HSQC of griseolutein D (**3**) (100 MHz, DMSO-*d*_6_)


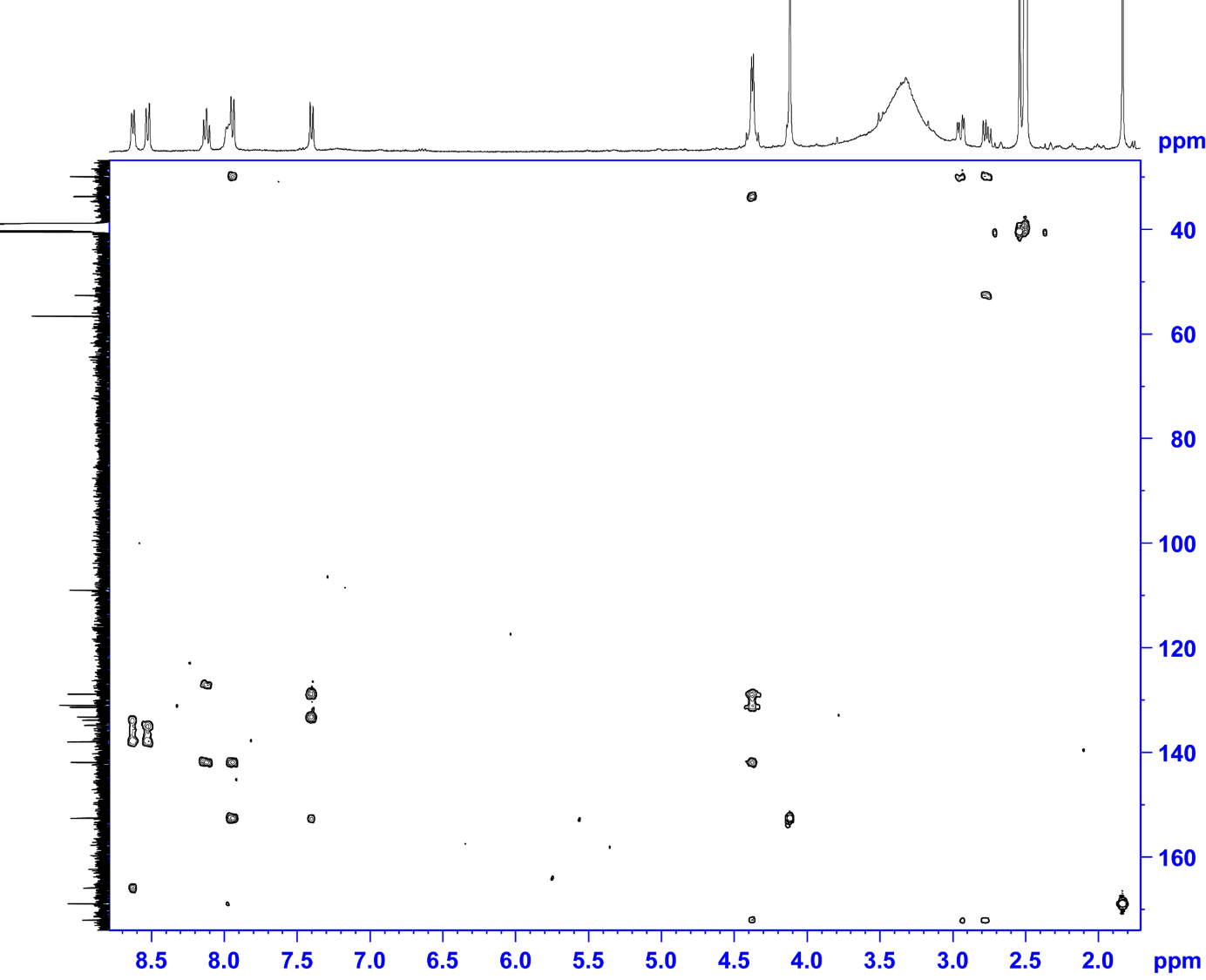
**Supplementary figure S17**

HMBC of griseolutein D (**3**) (100 MHz, DMSO-*d*_6_)


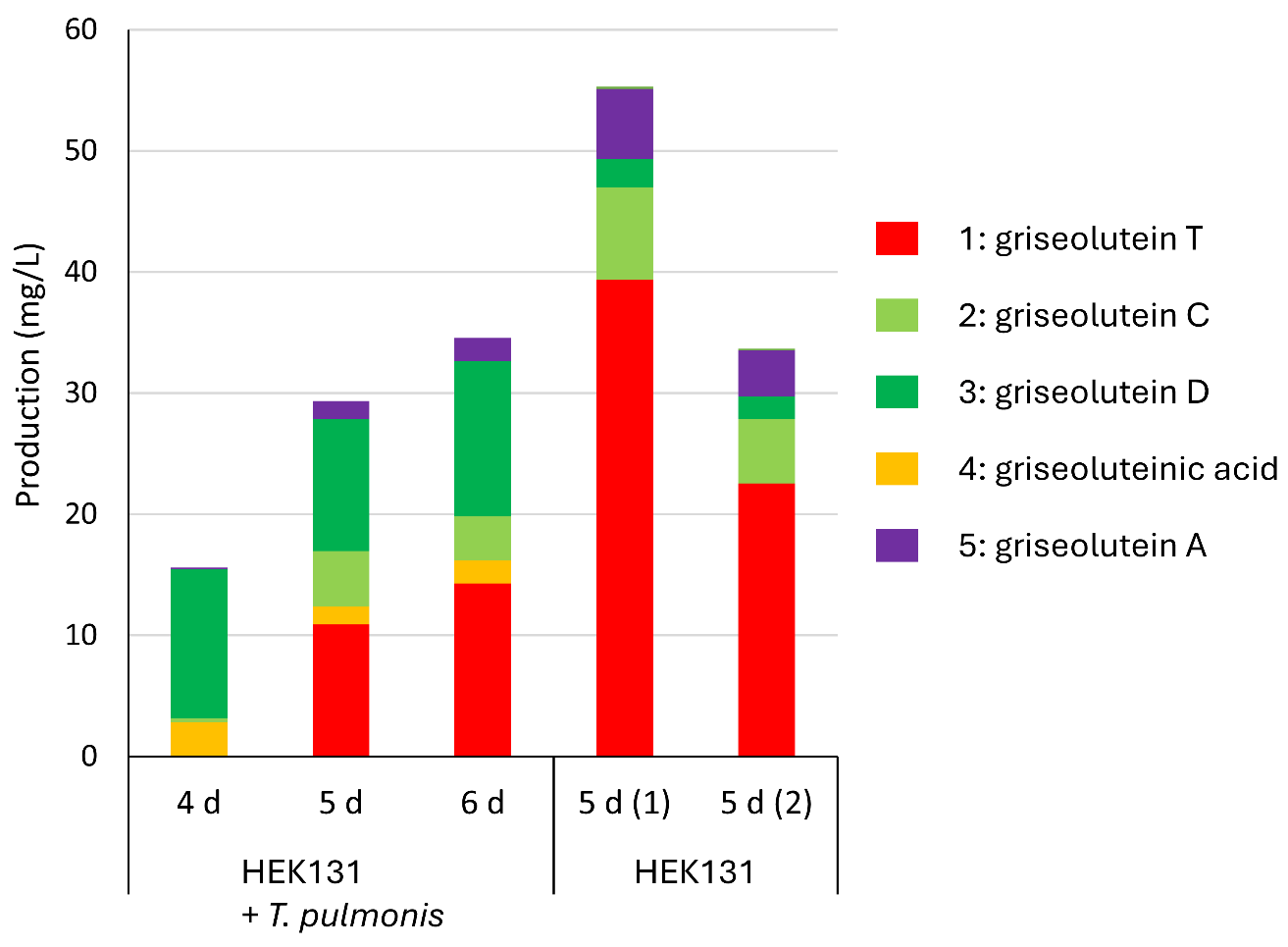


**Supplementary figure S18**

Comparison of production titers of compounds 1–5 in HEK131 monoculture and in combined-culture with *T. pulmonis*.


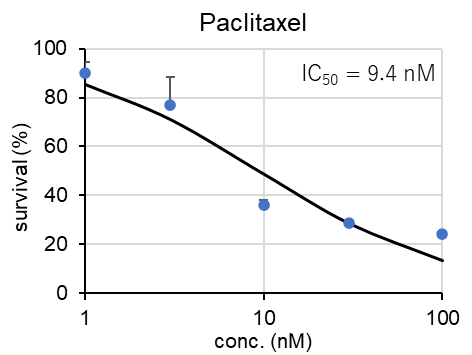

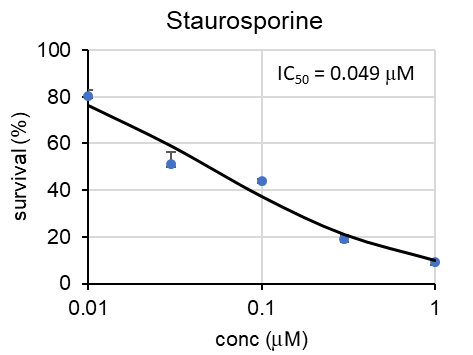

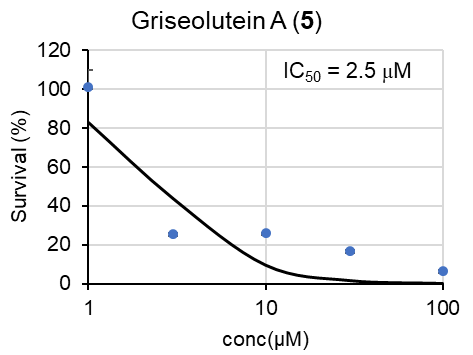

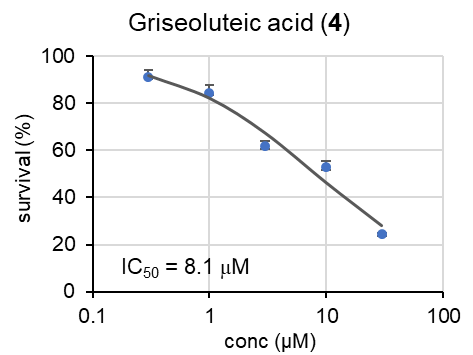

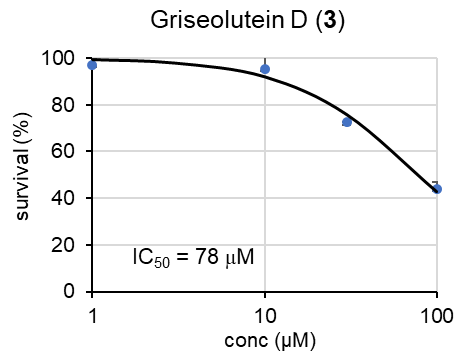

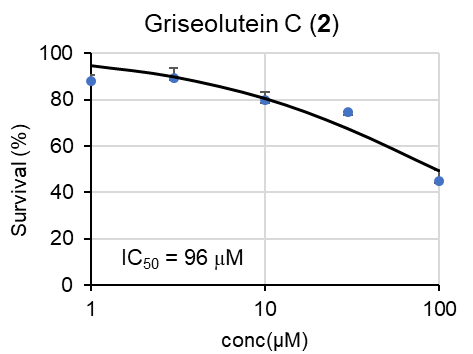

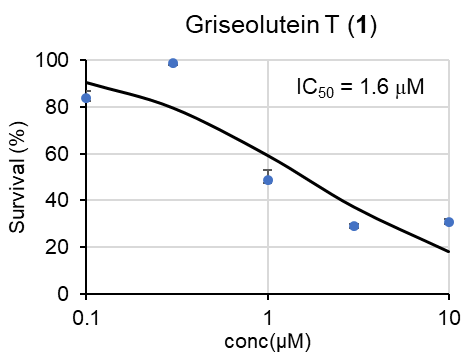
**Supplementary figure S19**

Cytotoxicity test. Staurosporine and paclitaxel were used as positive controls.

**Supplementary table S1**

^1^H NMR spectral data of compound **4** in DMSO-*d*_6_

|  | reported values | obs. values |
| --- | --- | --- |
| position | *δ*_H_ (J in Hz) | *δ*_H_ (J in Hz) |
| 2 | 8.694, d, (6.3) | 8.55, brd (8.0) |
| 3 | 8.145, dd, (7.0, 8.4) | 8.12, m |
| 4 | 8.551, d, (8.4) | 8.48, d (7.6) |
| 7 | 7.987, d, (7.7) | 7.97, d (8.0) |
| 8 | 7.480, d, (7.7) | 7.45, d (8.0) |
| COOH-11 | 15.543, br s | n.d. |
| 12 | 4.127, s | 4.12, s |
| 13 | 5.201, s | 5.20, s |
| OH-13 | 5.374, br s | n.d. |

^1^H NMR signals corresponding to positions COOH-11 and OH-13 were not detected (n.d.). The obs. ppm values were consistent with those published by Liu Y et al. *Journal of Natural Products*. 87(6):1540-1547. 2024 (doi: 10.1021/acs.jnatprod.4c00017).


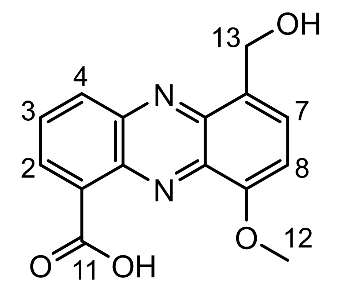


**Supplementary table S2**

List of the clinical isolates used for antibacterial tests and the MICs of griseolutein T (1) and known antibiotics against clinically isolated MRSA

| No | NUBL  no. | Area | Isolation year | MIC [mg L^-1^] | | | | | |
| --- | --- | --- | --- | --- | --- | --- | --- | --- | --- |
|  |  |  |  | 1 | VAN | FDX | TGC | MUP | PHZ-1-CA |
| 1 | 2104 | Tokyo | 2011 | 0.12 | 0.5 | 4 | 0.5 | 0.25 | >8 |
| 2 | 2105 | Tokyo | 2011 | 0.12 | 1 | 4 | 1 | 0.25 | >8 |
| 3 | 2106 | Saitama | 2011 | 0.12 | 0.5 | 2 | 0.25 | 0.12 | >8 |
| 4 | 2107 | Chiba | 2011 | 0.25 | 1 | 2 | 1 | 0.25 | >8 |
| 5 | 2109 | Fukushima | 2011 | 0.12 | 1 | 4 | 0.25 | >2 | >8 |
| 6 | 2110 | Fukushima | 2011 | 0.06 | 1 | 4 | 1 | >2 | >8 |
| 7 | 2111 | Fukushima | 2011 | 0.12 | 1 | 4 | 1 | >2 | >8 |
| 8 | 2113 | Wakayama | 2011 | 0.25 | 0.5 | 8 | 0.25 | 0.25 | >8 |
| 9 | 2115 | Kanagawa | 2011 | 0.25 | 2 | 4 | 1 | 0.5 | >8 |
| 10 | 2116 | Saitama | 2011 | 0.25 | 1 | 2 | 0.25 | 0.12 | >8 |
| 11 | 2117 | Chiba | 2011 | 0.06 | >8 | 2 | 2 | 0.25 | >8 |
| 12 | 2118 | Nagasaki | 2011 | 0.25 | 1 | 4 | 0.5 | 0.25 | >8 |
| 13 | 2119 | Aichi | 2011 | 0.25 | 0.5 | 4 | 2 | 0.25 | >8 |
| 14 | 2121 | Tokyo | 2011 | 0.25 | 1 | 2 | 0.5 | 0.25 | >8 |
| 15 | 2126 | Osaka | 2011 | 0.25 | 1 | 4 | 1 | 0.25 | >8 |
| 16 | 2127 | Gifu | 2011 | 0.12 | 1 | 4 | 0.25 | 0.12 | >8 |
| 17 | 2128 | Toyama | 2011 | 0.12 | 1 | 4 | 0.12 | 0.25 | >8 |
| 18 | 2130 | Nagano | 2011 | 0.12 | 1 | 1 | 0.25 | 0.12 | >8 |
| 19 | 2131 | Nagano | 2011 | 0.12 | 1 | 4 | 1 | 0.25 | >8 |
| 20 | 2135 | Aichi | 2011 | 0.12 | 1 | 2 | 1 | 0.25 | >8 |
| 21 | 2137 | Nagasaki | 2011 | 0.12 | 1 | 4 | 0.5 | 0.25 | >8 |
| 22 | 2142 | Okayama | 2011 | 0.06 | 1 | 2 | 1 | 0.25 | >8 |
| 23 | 2149 | Mie | 2011 | 0.12 | 1 | 4 | 1 | 0.25 | >8 |
| 24 | 2150 | Gunma | 2011 | 0.25 | 0.5 | 4 | 0.5 | 0.25 | >8 |
| 25 | 2152 | Osaka | 2011 | 0.06 | 1 | 4 | 1 | 0.25 | >8 |
| 26 | 2153 | Ishikawa | 2011 | 0.12 | 1 | 4 | 0.25 | 0.25 | >8 |
| 27 | 2154 | Toyama | 2011 | 0.12 | 1 | 4 | 0.25 | 0.25 | >8 |
| 28 | 2158 | Ibaraki | 2011 | 0.12 | 1 | 4 | 1 | 0.25 | >8 |
| 29 | 2165 | Tochigi | 2011 | 0.06 | 1 | 4 | 2 | 0.5 | >8 |
| 30 | 2167 | Wakayama | 2011 | 0.06 | 1 | 4 | 1 | 0.25 | >8 |

MIC: minimum inhibitory concentration_,_ MRSA: methicillin-resistant *Staphylococcus aureus*, NUBL: Nagoya University Bacteriology Laboratory, VAN: vancomycin, FDX: fidaxomicin, TGC: tigecycline, MUP: mupirocin, and PHZ-1-CA: phenazine-1-carboxylic acid (tubermycin B).

**Supplementary table S3**

List of the clinical isolates used for antibacterial tests and the MICs of griseolutein T (1) and known antibiotics against clinically isolated MSSA

| No | NUBL  no. | Area | Isolation year | MIC [mg L^-1^] | | | | | |
| --- | --- | --- | --- | --- | --- | --- | --- | --- | --- |
|  |  |  |  | 1 | VAN | FDX | TGC | MUP | PHZ-1-CA |
| 1 | 26 | Nagano | 2010 | 0.06 | 0.5 | 8 | 0.12 | 0.25 | >8 |
| 2 | 34 | Aichi | 2010 | 0.12 | 1 | 8 | 0.12 | 0.25 | >8 |
| 3 | 38 | Kanagawa | 2010 | 0.12 | 1 | 2 | 0.25 | 0.25 | >8 |
| 4 | 40 | Saitama | 2010 | 0.03 | 1 | 1 | 0.06 | 0.06 | >8 |
| 5 | 41 | Ibaraki | 2010 | 0.25 | 1 | 4 | 0.12 | 0.25 | >8 |
| 6 | 42 | Toyama | 2010 | 0.12 | 1 | 4 | 0.25 | 0.25 | >8 |
| 7 | 47 | Osaka | 2010 | 0.25 | 0.5 | 8 | 0.25 | 0.25 | >8 |
| 8 | 48 | Tokyo | 2010 | 0.06 | 1 | 8 | 0.25 | 0.25 | >8 |
| 9 | 49 | Tochigi | 2010 | 0.25 | 0.5 | 4 | 0.25 | 0.25 | >8 |
| 10 | 50 | Wakayama | 2010 | 0.06 | 1 | 8 | 0.25 | 0.25 | >8 |
| 11 | 62 | Chiba | 2011 | 0.25 | 1 | 4 | 0.12 | 0.25 | >8 |
| 12 | 65 | Aomori | 2011 | 0.12 | 0.5 | 4 | 0.12 | 0.25 | >8 |
| 13 | 66 | Hiroshima | 2011 | 0.25 | 0.5 | 4 | 0.25 | 0.25 | >8 |
| 14 | 68 | Shizuoka | 2011 | 0.25 | 2 | 8 | 0.25 | 0.25 | >8 |
| 15 | 77 | Ishikawa | 2011 | 0.12 | 1 | 8 | 0.12 | 0.12 | >8 |
| 16 | 94 | Nagano | 2011 | 0.25 | 1 | 8 | 0.25 | 0.25 | >8 |
| 17 | 101 | Fukui | 2011 | 0.12 | 0.5 | 8 | 0.25 | 0.12 | >8 |
| 18 | 113 | Yamanashi | 2011 | 0.25 | 0.5 | 8 | 0.25 | 0.25 | >8 |
| 19 | 121 | Gifu | 2011 | 0.12 | 1 | 8 | 0.25 | 0.25 | >8 |
| 20 | 27 | Nagano | 2010 | 0.25 | 1 | 4 | 0.5 | 0.25 | >8 |
| 21 | 31 | Nagano | 2010 | 0.25 | 1 | 4 | 0.25 | 0.25 | >8 |
| 22 | 32 | Nagano | 2010 | 0.25 | 1 | 4 | 0.25 | 0.25 | >8 |
| 23 | 33 | Nagano | 2010 | 0.25 | 1 | 8 | 0.25 | 0.25 | >8 |
| 24 | 35 | Aichi | 2010 | 0.25 | 1 | 8 | 0.25 | 0.25 | >8 |
| 25 | 36 | Nagano | 2010 | 0.5 | 1 | 8 | 0.25 | 0.25 | >8 |
| 26 | 37 | Aichi | 2010 | 0.06 | 1 | 4 | 0.25 | 0.25 | >8 |
| 27 | 39 | Nagano | 2010 | 0.06 | 1 | 8 | 0.25 | 0.25 | >8 |
| 28 | 28 | Nagano | 2010 | 0.03 | 1 | 8 | 0.25 | 0.25 | >8 |
| 29 | 29 | Nagano | 2010 | 0.06 | 0.5 | 4 | 0.25 | 0.25 | >8 |
| 30 | 30 | Nagano | 2010 | 0.25 | 1 | 8 | 0.5 | 0.25 | >8 |

MIC: minimum inhibitory concentration_,_ MSSA: methicillin-susceptible *Staphylococcus aureus*, NUBL: Nagoya University Bacteriology Laboratory, VAN: vancomycin, FDX: fidaxomicin, TGC: tigecycline, MUP: mupirocin, and PHZ-1-CA: phenazine-1-carboxylic acid (tubermycin B).

**Supplementary table S4**

List of the clinical isolates used for antibacterial tests and the MICs of griseolutein T (1) and known antibiotics against clinically isolated VRE

| No | NUBL  no. | Area | Isolation year | MIC [mg L^-1^] | | | | | |
| --- | --- | --- | --- | --- | --- | --- | --- | --- | --- |
|  |  |  |  | 1 | VAN | FDX | TGC | MUP | PHZ-1-CA |
| 1 | 9462 | Fukuoka | 2012 | 0.5 | 1 | 2 | 0.06 | 0.5 | >8 |
| 2 | 9463 | Fukuoka | 2012 | 0.5 | 1 | 1 | 0.06 | 0.5 | >8 |
| 3 | 9469 | Chiba | 2013 | 1 | >16 | 2 | 0.06 | 0.5 | >8 |
| 4 | 10193 | Chiba | 2014 | 1 | 16 | 2 | 0.06 | 0.5 | >8 |
| 5 | 11580 | Saitama | 2014 | 0.12 | 8 | 2 | 0.06 | 0.5 | >8 |
| 6 | 12464 | Kagawa | 2014 | 1 | >16 | 2 | 0.03 | 0.5 | >8 |
| 7 | 12466 | Hyogo | 2014 | 0.5 | >16 | 1 | 0.03 | 0.5 | >8 |
| 8 | 12467 | Kagawa | 2014 | 0.12 | 16 | 2 | 0.06 | 0.5 | >8 |
| 9 | 14481 | Osaka | 2014 | 0.03 | 1 | 1 | 0.03 | 0.5 | >8 |
| 10 | 14490 | Kagawa | 2014 | 0.12 | 16 | 2 | 0.06 | 0.5 | >8 |
| 11 | 14491 | Hyogo | 2014 | 0.03 | 16 | 0.5 | 0.03 | 0.5 | >8 |
| 12 | 14492 | Kagawa | 2014 | 0.12 | 16 | 2 | 0.06 | 0.5 | >8 |
| 13 | 14495 | Osaka | 2014 | 0.03 | 1 | 1 | 0.03 | 0.5 | >8 |
| 14 | 14496 | Osaka | 2014 | 0.03 | 1 | 1 | 0.03 | 0.5 | >8 |
| 15 | 14497 | Osaka | 2014 | 0.03 | 1 | 1 | 0.03 | 0.25 | >8 |
| 16 | 14498 | Fukuoka | 2014 | 1 | 2 | 2 | 0.06 | 0.5 | >8 |
| 17 | 14499 | Fukuoka | 2014 | 1 | 2 | 2 | 0.06 | 0.5 | >8 |
| 18 | 14500 | Kagawa | 2014 | <0.016 | 0.5 | 0.5 | 0.06 | 0.25 | 2 |
| 19 | 22226 | Kanagawa | 2016 | 0.12 | >16 | 2 | 0.06 | 0.5 | >8 |
| 20 | 23202 | Okayama | 2017 | 0.12 | 16 | 2 | 0.06 | 0.5 | >8 |
| 21 | 23551 | Tokyo | 2016 | 0.03 | 4 | 2 | 0.12 | 0.5 | >8 |
| 22 | 23552 | Tokyo | 2017 | 0.12 | >16 | 2 | 0.06 | 0.5 | >8 |
| 23 | 23553 | Tokyo | 2017 | 0.06 | 4 | 2 | 0.06 | 0.5 | >8 |
| 24 | 23554 | Tokyo | 2017 | 0.06 | 16 | 2 | 0.06 | 0.5 | >8 |
| 25 | 23558 | Okayama | 2017 | 0.25 | 16 | 2 | 0.12 | 0.25 | >8 |
| 26 | 23559 | Okayama | 2017 | 0.06 | 8 | 2 | 0.06 | 0.5 | >8 |
| 27 | 23561 | Kanagawa | 2017 | 0.06 | >16 | 2 | 0.06 | 0.5 | >8 |

MIC: minimum inhibitory concentration_,_ VRE: vancomycin-resistant enterococci, NUBL: Nagoya University Bacteriology Laboratory, VAN: vancomycin, FDX: fidaxomicin, TGC: tigecycline, MUP: mupirocin, and PHZ-1-CA: phenazine-1-carboxylic acid (tubermycin B).

**Supplementary table S5**

List of the clinical isolates used for antibacterial tests and the MICs of griseolutein T (1) and known antibiotics against clinically isolated *C. difficile*

| No | NUBL  no. | Area | Isolation year | MIC [mg L^-1^] | | | | | |
| --- | --- | --- | --- | --- | --- | --- | --- | --- | --- |
|  |  |  |  | 1 | VAN | FDX | TGC | MUP | PHZ-1-CA |
| 1 | 3824 | Aichi | 2010 | <0.016 | 0.25 | <0.016 | <0.016 | >8 | 8 |
| 2 | 3835 | Tochigi | 2010 | 0.03 | 0.25 | <0.016 | <0.016 | >8 | 8 |
| 3 | 3838 | Ibaraki | 2010 | 0.06 | 0.25 | <0.016 | <0.016 | >8 | 8 |
| 4 | 3839 | Kumamoto | 2010 | 0.06 | 0.25 | <0.016 | <0.016 | >8 | 8 |
| 5 | 3845 | Tokyo | 2010 | 0.06 | 0.25 | <0.016 | <0.016 | >8 | 8 |
| 6 | 3846 | Kanagawa | 2010 | 0.06 | 0.25 | <0.016 | <0.016 | >8 | 8 |
| 7 | 3848 | Chiba | 2010 | 0.06 | 0.25 | <0.016 | <0.016 | >8 | 8 |
| 8 | 3853 | Osaka | 2010 | <0.016 | 0.12 | <0.016 | <0.016 | >8 | 8 |
| 9 | 4802 | Gifu | 2010 | 0.06 | 0.25 | <0.016 | <0.016 | >8 | 4 |
| 10 | 4807 | Mie | 2010 | 0.06 | 0.12 | <0.016 | <0.016 | >8 | 8 |
| 11 | 4818 | Ishikawa | 2010 | 0.06 | 0.25 | <0.016 | <0.016 | >8 | 4 |
| 12 | 4824 | Saitama | 2011 | 0.06 | 0.25 | <0.016 | <0.016 | >8 | 4 |
| 13 | 4837 | Aomori | 2012 | 0.06 | 0.25 | <0.016 | <0.016 | >8 | 4 |
| 14 | 13382 | Yamagata | 2014 | 0.06 | 0.25 | <0.016 | <0.016 | >8 | 8 |
| 15 | 13377 | Chiba | 2014 | 0.06 | 0.12 | <0.016 | <0.016 | >8 | 4 |
| 16 | 13381 | Yamanashi | 2014 | 0.06 | 0.25 | <0.016 | <0.016 | >8 | 8 |
| 17 | 13383 | Yamagata | 2014 | 0.06 | 0.25 | <0.016 | <0.016 | >8 | 8 |

MIC: minimum inhibitory concentration_,_ *C. difficile*: *Clostridioides difficile*, NUBL: Nagoya University Bacteriology Laboratory, VAN: vancomycin, FDX: fidaxomicin, TGC: tigecycline, MUP: mupirocin, and PHZ-1-CA: phenazine-1-carboxylic acid (tubermycin B).

**Supplementary table S6**

List of the type strains used for antibacterial tests and the MICs of griseolutein T (1) and known antibiotics against type strains

|  | MIC [mg L^-1^] | | | | | |
| --- | --- | --- | --- | --- | --- | --- |
|  | **1** | **VAN** | **FDX** | **TGC** | **MUP** | **PHZ-1-CA** |
| **MRSA**  **ATCC43300** | 0.06 | 1 | 4 | 0.25 | 0.25 | >8 |
| **MSSA**  **ATCC29213** | 0.25 | 2 | 4 | 0.25 | 0.25 | >8 |
| **VRE**  **ATCC51299** | 2 | >16 | 2 | 0.06 | >8 | >8 |
| ***C. difficile* ATCC700057** | 0.03 | 0.25 | <0.016 | 0.03 | >8 | 4 |

MIC: minimum inhibitory concentration_,_ MRSA: methicillin-resistant *Staphylococcus aureus*, MSSA: methicillin-susceptible *Staphylococcus aureus*, VRE: vancomycin-resistant enterococci, *C. difficile*: *Clostridioides difficile,* VAN: vancomycin, FDX: fidaxomicin, TGC: tigecycline, MUP: mupirocin, and PHZ-1-CA: phenazine-1-carboxylic acid (tubermycin B).

**Supplementary table S7**

Relative ion intensity of isotope peaks for 2 and 3

| compounds | ion composition | rel. intensity (%) | | |
| --- | --- | --- | --- | --- |
|  |  | MH | MH+1 | MH+2 |
| griseolutein C (**2**) | obs (M+H)^+^ | 100 | 22.4 | 7.6 |
|  | cal (C_20_H_20_N_3_O_6_S) | 100 | 20.5 | 4.5 |
|  | cal (C_20_H_20_N_3_O_8_) | 100 | 20.5 | 2 |
| griseolutein D (**3**) | obs (M+H)^+^ | 100 | 21 | 6.9 |
|  | cal (C_19_H_18_N_3_O_6_**S**) | 100 | 21.6 | 4.5 |
|  | cal (C_19_H_18_N_3_O_8_) | 100 | 21.6 | 2.2 |
